# Evolutionary genomics of *Culex pipiens*: global and local adaptations associated with climate, life-history traits and anthropogenic factors

**DOI:** 10.1098/rspb.2015.0728

**Published:** 2015-07-07

**Authors:** Hosseinali Asgharian, Peter L. Chang, Sergey Lysenkov, Victoria A. Scobeyeva, William K. Reisen, Sergey V. Nuzhdin

**Affiliations:** 1 Program in Molecular and Computational Biology, University of Southern California, Los Angeles, CA 90089, USA; 2 Department of Evolution, Moscow State University, Moscow 119991, Russia; 3 Center for Vectorborne Diseases, Department of Pathology, Microbiology and Immunology, School of Veterinary Medicine, University of California, Davis, CA 95616, USA; 4 St. Petersburg State Polytechnical University, Sanct Petersburg, Russia

**Keywords:** *Culex pipiens*, selective sweeps, histones, principal component analysis, population structure, differential selection

## Abstract

We present the first genome-wide study of recent evolution in *Culex pipiens* species complex focusing on the genomic extent, functional targets and likely causes of global and local adaptations. We resequenced pooled samples of six populations of *C. pipiens* and two populations of the outgroup *Culex torrentium*. We used principal component analysis to systematically study differential natural selection across populations and developed a phylogenetic scanning method to analyse admixture without haplotype data. We found evidence for the prominent role of geographical distribution in shaping population structure and specifying patterns of genomic selection. Multiple adaptive events, involving genes implicated with autogeny, diapause and insecticide resistance were limited to specific populations. We estimate that about 5–20% of the genes (including several histone genes) and almost half of the annotated pathways were undergoing selective sweeps in each population. The high occurrence of sweeps in non-genic regions and in chromatin remodelling genes indicated the adaptive importance of gene expression changes. We hypothesize that global adaptive processes in the *C. pipiens* complex are potentially associated with South to North range expansion, requiring adjustments in chromatin conformation. Strong local signature of adaptation and emergence of hybrid bridge vectors necessitate genomic assessment of populations before specifying control agents.

## 1. Introduction

Arms races between antagonistic species have been of longstanding interest to evolutionary biologists [1,2]. Humans have developed unique ways of fighting against competitors, parasites and their vectors through the use of scientific and technological innovations such as application of antibiotics and pesticides, living in artificially designed cities with high hygienic standards and avoidance of high disease-transmission-risk behaviours. The race is, however, far from over, as emphasized by the emergence of new virulent pathogenic strains, evolution of insecticide-resistant vectors and the adaptations of several pathogenic or competitor species for living in cities. Notably, any such adaptation takes place locally at first, even if it does spread to gain global significance eventually.

Here, we present the results of the first genome-wide analysis of population structure and recent natural selection in the *Culex pipiens* complex, members of which are notorious vectors of West Nile Virus, St. Louise Encephalitis Virus and filariasis worms in the US and worldwide [3–5]. This complex consists of *Culex quinquefasciatus* (the southern house mosquito) and *C. pipiens* (the northern house mosquito). Two biological forms (biotypes) *C. pipiens* f. pipiens and *C. pipiens* f. molestus have been described within the *C. pipiens* species based on physiological and ecological differences including the choice of host species, seasonal activity, mating behaviour and preferred habitat [3,6,7]. Hybridization between these two forms and also with *C. quinquefasciatus* has been reported in certain areas, leading to the rise of bridge vectors transmitting pathogens between birds and humans [3,6,8–10].

We studied six *C. pipiens* populations and two populations of the closely related species *Culex torrentium* as an outgroup living within or close to human-inhabited areas in Europe and North America (Moscow and Aleksin, Russia and Sacramento, CA, USA), aiming to investigate two fundamental population genetic aspects of *C. pipiens*: population structure and natural selection. On population structure, we asked: (i) Does geography, habitat type or biological form mainly determine the organization of genetic variation in *Culex*? (ii) Do genomic data support genetic isolation and imminent speciation of pipiens and molestus forms, or conversely, do we detect considerable admixture between them? On the matter of natural selection, we asked: (i) Which genes and biological functions are the targets of recent selective sweeps? (ii) To what degree do protein sequence alterations and gene expression changes contribute to adaptation? (iii) What factors are the likely causes of recent sweeps? (iv) Are recent adaptations happening congruently or otherwise in different populations?

## 2. Material and methods

### (a) Mosquito samples

Mosquito samples were taken from urban and suburban areas in Sacramento (CA, USA), Moscow and Aleksin (Central Russia) (table 1).

**Table 1.** Samples used in this study and their average genome-wide variability statistics.

sample	location	habitat	taxonomical identification	no. pooled individuals	average *π*^a^	average *θ*^a^
A1	Aleksin	urban	*C. pipiens* f. molestus	224	0.01937	0.02030
A4	Aleksin	suburban	*C. pipiens* f. pipiens	132	0.02403	0.02531
M1	Moscow	urban	*C. pipiens* f. molestus	26	0.01821	0.01905
M2^b^	Moscow	suburban	*C. torrentium*	28	0.01933	0.02070
M4^b^	Moscow	suburban	*C. torrentium*	195	0.01740	0.01820
S1	Sacramento	urban (males)	*C. pipiens* f. molestus	15	0.02291	0.02354
S2^b^	Sacramento	suburban (males)	*C. pipiens*, mixed molestus and pipiens forms	13	0.02347	0.02438
S3^b^	Sacramento	suburban (females)	*C. pipiens*, mixed molestus and pipiens forms	64	0.02276	0.02365

^a^Average of 10 kb sliding windows. Only positions covered 4–40× were included.

^b^The two Moscow suburban samples and the two Sacramento suburban samples were each caught independently at different sites and represent different populations.

### (b) Sequencing and mapping to the reference

Genomic DNA was extracted from the pool of mosquitoes collected from each of the eight populations, prepared into separate libraries and sequenced as paired-end 101 bp reads on an Illumina HiSeq machine. Sequenced reads were aligned as pairs using BWA 0.5.7 [11] to the complete *C. quinquefasciatus* draft genome downloaded from the Broad Institute (see https://www.broadinstitute.org/annotation/genome/culex_pipiens.4/MultiDownloads.html). Reads were allowed up to 12 mismatches throughout the 101 bp per end; they were mapped to the genome and those that did not map uniquely were filtered out. All other BWA alignment parameters were set to default values.

### (c) Population genetic analyses

The reads mapping to the mitochondrial cytochrome oxidase subunit I gene (COI) and to the CQ11 microsatellite locus were used to ascertain species and biotype identities of the populations, respectively [12,13].

*F*_st_ was calculated for 10 kb sliding windows between each pair of populations according to the methods in [14,15] and averaged across the genome. Maximum-likelihood phylogenetic trees were constructed from sliding windows of non-overlapping 10 kb using RAxML [16]. A custom Python code was used to calculate the percentage of time each two populations were nearest neighbours on the tree. Principal component analysis (PCA) of allele frequencies was done on biallelic positions with coverage 4–40×.

The software package Popoolation v. 1.2.2 was used to estimate measures of variation (*π* and *θ*) and Tajima's *D* from the pooled sequence data [17]. Only positions with coverage 4–40× were used and the minimal legitimate count for the minor allele was set to 2. Synonymous (syn) and non-synonymous (nsyn) polymorphisms were assigned using the same software and the .gff file downloaded from the Broad Institute website.

To detect selective sweeps, we first obtained the allele frequency spectrum (AFS) from the whole genome as the neutral background, and then tried to identify a certain form of skewness in AFS of linked sites, typically associated with selective sweeps [18,19]. The approach in [18,19] has been modified to apply to pooled sequence data [20] and incorporated into the software package Pool-hmm [21]. We ran Pool-hmm in two steps. First, AFS was built based on the whole genome for each sample with coverage 4–40×, *θ* = 0.02 (based on the Popoolation output, see table 1) and sampling ratio of 20 (5% of positions were used for estimation of AFS). Second, sweep regions were detected separately for each supercontig with the same coverage range as above and transition probability of *k* = 1 × 10^−6^ based on the AFS created in the previous step. PCA of Pool-hmm sweep scores was done to compare the broad patterns of genomic selection among the studied populations. Each gene was treated as an observation point and each sample label as an initial variable. We used linear regression to check for potential biases introduced by sequencing coverage variation in calculation of Tajima's *D* and Pool-hmm scores.

To understand the nature of mutations associated with the sweep events, we did a case study focusing on a 137-kb block (*C. quinquefasciatus* genome supercontig 3.392: 626-137726) consisting exclusively of 80 histone genes including multiple paralogues of each histone type (H1, H2A, H2B, H3 and H4). The large number of polymorphic sites in H1 genes allowed statistical analysis of association of different types of amino acid changes with certain structural features of the protein. For the polymorphic positions, we cross-examined three structural attributes (domain, secondary structure and solvent accessibility) with three biochemical aspects (addition or removal of proline, the charge difference between the two amino acids, and addition or removal of serine or threonine) using independence tests (*χ*^2^ and Fisher's exact). Electrostatic interactions are important in forming the three-dimensional structure of the protein, as well as its DNA-binding function. The reason to include the Ser/Thr category was that they are targets of phosphorylation, which is the best-studied epigenetic modification of H1 [22], although several other types of modification are also reported [23]. We included Pro because, apart from its structural peculiarities, we found tremendous excess conversion from other amino acids in the reference genome to Pro at polymorphic positions (electronic supplementary material, S1).

We examined the functional significance of the sweep genes using three different approaches: Gene Ontology (GO) enrichment analysis, Kyoto Encyclopedia of Genes and Genomes (KEGG; see http://www.genome.jp/kegg/) pathway annotations and checking the sweep status of genes with experimentally verified functions reported in the literature. GO enrichment analysis on targets of selection was performed using the online software GOEAST [24]. GO annotations for *Culex* genes were downloaded from vectorbase.org/biomart. The complete annotation file was used as the default background set. For each population, two enrichment tests were run with different selected gene sets: (i) 200 genes (approx. 1% of the total number of genes in the genome) with highest Pool-hmm scores, and (ii) 200 genes with lowest Tajima's *D* values. Enrichments with false discovery rate < 0.1 were considered significant. An ANOVA test was done to make sure that gene length did not bias GO enrichment results (electronic supplementary material, S1). The annotated pathways for *C. quinquefasciatus* were downloaded from the KEGG Pathway database [25]. For each pathway, maximum and average sweep scores were determined and the number of genes with sweep score more than 4 was counted. The number of pathways in which each gene functioned was counted as a proxy for multifunctionality (related to the concept of pleiotropy).

### (d) Statistical procedures

All statistical analyses including calculation of descriptive statistics, correlations, independence tests, regressions, ANOVA and PCA were done using SAS v. 9.3 and SAS JMP Pro v. 10.0.0.

More details on the methods can be found in electronic supplementary material, S1.

## 3. Results

### (a) Diversity depends on biotype but population structure is shaped by geography

Whole-genome Illumina resequencing of the eight individually pooled populations resulted in 42× total coverage of the *C. quinquefasciatus* draft genome, identifying 6.7 M segregating sites among these populations. With more than 461 MB covered by Illumina sequences, this equated to roughly one segregating allele every 69 nucleotides. Table 1 shows average *π* and *θ* for 10 kb sliding windows across the genomes of the eight samples. The divergence of our *C. pipiens* populations from the *C. quinquefasciatus* reference genome ranged 0.6–1.8%. As expected, *C. torrentium* populations were more divergent (2.8–3.3%). In accordance with this, about 60–75% and 41–46% of total Illumina reads from *C. pipiens* and *C. torrentium* samples mapped onto the reference genome, respectively. The average sequencing depth across the whole covered segments of the genomes was 2–8× for our samples (the lowest ones belonging to *C. torrentium*). However, we only included the positions with coverages 4–40× in estimation of diversity and selection metrics. The average coverage across those positions in genic regions (whose output was fed into the PCA) ranged 8–19×. Only 7.3% and 5.4% of variance in the observed values of Tajima's *D* and Pool-hmm score for genes could be explained by coverage differences, respectively (linear regression *R*^2^, *p* < 0.0001). PCA on gene coverages did not produce any patterns similar to the geography-dependent clustering observed for the selection metrics. Thus, coverage did not seem to bias the calculation of population parameters significantly.

Differentiation among populations depended on geographical distance demonstrated by 10 kb sliding window genomic scans of *F*_st_ and phylogenetic tree structure (table 2). As expected, the largest distances belonged to *C. torrentium* versus *C. pipiens* comparisons. Within the *C. pipiens* complex, population structure corresponded to geographical proximity for both Russian and American samples. Neither *F*_st_ nor phylogenies suggested clustering based on habitat type (urban versus suburban) or biological form (molestus versus pipiens). PCA of allele frequencies mirrored this image (electronic supplementary material, S2). The reference sequence (*C. quinquefasciatus*) clustered closely with two *C. pipiens* samples only: A1 and S1 (table 2). The shared genomic regions contributing to this closeness, therefore, seem likely to have originated from recent local admixture rather than ancestral shared polymorphisms between *C. pipiens* and *C. quinquefasciatus*.

**Table 2.** Population structure in the eight samples demonstrated through pairwise *F*_st_ values and phylogenetic frequency of neighbourhood. The lower half of the table reports the average *F*_st_ of 10 kb sliding windows in pairwise comparisons. The upper half shows in what percentage of the phylogenetic trees based on 10 kb windows each two populations are nearest neighbours.

	A1	A4	M1	M2	M4	S1	S2	S3	Ref
A1	−	15.60	12.16	0.64	0.80	7.68	7.83	8.54	8.07
A4	0.166	−	12.90	2.24	1.50	5.19	6.05	6.66	3.90
M1	0.211	0.211	−	1.32	1.10	8.66	10.59	13.05	3.41
M2	0.487	0.457	0.497	−	78.23	0.58	0.77	0.58	2.55
M4	0.502	0.461	0.499	0.144	−	1.14	0.71	0.58	4.97
S1	0.193	0.219	0.241	0.451	0.460	−	15.29	15.72	9.18
S2	0.176	0.201	0.228	0.430	0.427	0.143	−	18.61	5.86
S3	0.160	0.187	0.217	0.414	0.402	0.143	0.138	−	3.56

### (b) Positive selection acts on non-coding and coding regions with non-synonymous mutations playing an important role

About 50–65% of the regions targeted by Pool-hmm with high confidence scores in *C. pipiens* populations coincided with coding sequences of annotated genes. Scanning the genome in 10 kb sliding windows, we found that the windows containing genic sequences were generally more likely to overlap with a sweep region (odds ratio = 1.19, independence *χ*^2^ test, *p* < 0.0001). In all of the *C. pipiens* samples, the total number of coding sequence polymorphisms per gene, as well as the number of either syn or nsyn polymorphisms, was smaller in sweep regions compared with the rest of the genome, compatible with the purported reduced variation around the sweep site (table 3). On the other hand, the ratio of nsyn/syn sites was always higher in the sweep regions. In *C. torrentium* samples, the trends were not exactly similar. The higher nsyn/syn ratio was still true; however, the correlation of each type of polymorphism with sweep status was either non-existent or slightly positive.

**Table 3.** Coding sequence polymorphisms within and outside sweep regions. Sweep status 0: gene resides in a region not detected by Pool-hmm or detected with a score of less than 4; sweep status 1: gene resides in a region detected by Pool-hmm with a score of greater than or equal to 4; *N*: number of genes; total: genewise average of all polymorphisms in the coding DNA sequence; syn: genewise average of synonymous polymorphisms; nsyn: genewise average of non-synonymous polymorphisms; values in the correlation columns represent Spearman partial correlation coefficients controlled for gene length; n.s.: correlation not significant at *p* = 0.05; figures in parentheses: *p*-value of the correlation (*p*-value less than 0.0001 where not stated). Repeating the analysis with sweep scores greater than or equal to 2 or 8 as the cut-off (instead of 4) yielded very similar results (not shown). In calculation of the ratio, 0.5 was added to both syn and nsyn counts to avoid division by zero.

	genes with sweep status 0				genes with sweep status 1				correlation with sweep status 1			
sample	*N*	total	syn	nsyn	*N*	total	syn	nsyn	total	syn	nsyn	nsyn/syn ratio
A1	19 120	43.52	30.00	13.52	1186	17.50	7.94	9.56	−0.1574	−0.1985	−0.0743	0.2147
A4	19 129	40.91	29.27	11.64	1177	22.51	11.81	10.70	−0.1210	−0.1634	−0.0263	0.1894
M1	16 114	44.54	27.42	17.12	4192	29.90	14.93	14.97	−0.1896	−0.2407	−0.0990	0.2441
M2	18 376	26.48	15.03	11.44	1930	34.75	18.72	16.03	0.0330	0.0157 (0.0252)	0.0563	0.0622
M4	19 260	9.70	5.77	3.93	1046	10.23	5.81	4.42	n.s.	n.s.	0.0256 (0.0003)	0.0202 (0.0040)
S1	17 713	39.48	28.62	10.87	2593	19.95	11.06	8.89	−0.2221	−0.2748	−0.0934	0.2655
S2	18 055	62.80	42.74	20.06	2251	30.11	16.29	13.81	−0.2390	−0.2828	−0.1336	0.2647
S3	17 510	61.82	40.12	21.70	2796	30.39	16.45	13.94	−0.2766	−0.3107	−0.1852	0.2478

A summary of Tajima's *D* values and Pool-hmm scores can be found in electronic supplementary material, S3.

### (c) Positive selection acts on a wide variety of biological functions in the *Culex* genome from chromatin organization to insecticide resistance

Table 3 shows the numbers of genes detected as sweep targets in each population. Based on both Pool-hmm and Tajima's *D* data, the most commonly enriched GO terms were related to chromatin and nucleosome structure and modification (electronic supplementary material, S4). The list of genes contributing to these terms included histones and chromatin remodelling factors (not shown). This was surprising given the well-known conservation of histone sequences, and motivated us to investigate the likely causes of selective sweeps in histones from a structure–function perspective in more detail (§§3*e* and 4*e*). Gene length proved not to be a significant confounder in the GO enrichment analysis (electronic supplementary material, S4).

Examination of the Pool-hmm results in the context of the functional pathways annotated for *C. quinquefasciatus* in the KEGG database demonstrated two important points: first, in every one of the eight populations more than half of the 129 pathways were affected by positive selection as they contained at least one gene with a sweep score more than 4. These pathways encompassed a large variety of functions including but not limited to amino acid biosynthesis, glycosphingolipid biosynthesis, signalling pathways (Notch, Jak-STAT and MAPK) and dorsoventral axis formation. Second, the number of pathways a gene functioned in was not correlated with the number of populations it was selected in nor the strength of selection when it happened.

Finally, we compiled a list of genes that have been shown by gene expression or mutant phenotyping studies to affect specific life-history traits of *Culex* (such as diapause, autogeny and mating behaviour), confer insecticide resistance or facilitate adaptation to temperature fluctuations. Histones and chromatin remodelling factors, ribosomal proteins, members of the P450 family, chaperonins and heat-shock proteins, vitellogenins and vitellogenin convertase, cadherins, superoxide dismutases and salivary proteins were notable genes with experimentally verified functional roles that were undergoing sweeps in multiple populations (electronic supplementary material, S4, p. 10).

### (d) Many specific adaptations in the *Culex pipiens* genome happen locally

**Figure 1.**
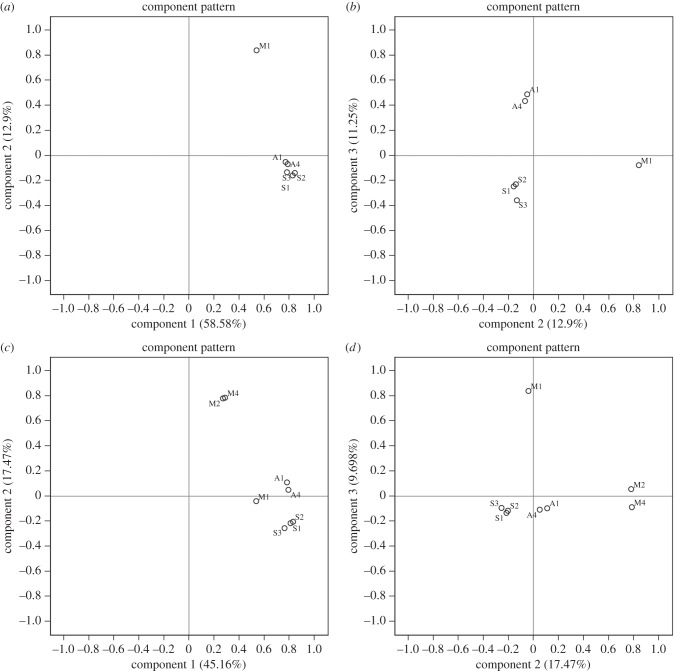
The first three PCs of Pool-hmm scores of genes from *C. pipiens* samples (*a*,*b*) or *C. pipiens* and *C. torrentium* together (*c*,*d*).

For both Pool-hmm and Tajima's *D*, we performed PCA once with the six *C. pipiens* samples only, and a second time with all of eight *Culex* samples included. The first principal component (PC) was always highly correlated with all of the sample labels and did not separate the samples from each other decisively (figure 1*a*,*c*). In the case of the six *C. pipiens* samples, the second and third PCs demonstrated the local nature of adaptation in the most conspicuous way, producing three distinct clusters containing the samples from Moscow, Aleksin and Sacramento (figure 1*b*). Including all eight samples, the combination of the second and the third components produced four clusters: one for the *C. torrentium* samples and three for each of the *C. pipiens* locations (figure 1*d*). The results of PCA on Tajima's *D* values were very similar (not shown).

### (e) Evolution of the conserved: parallel adaptation of histones in *Culex pipiens* and *Culex torrentium*

The 137 kb histone block that we examined closely showed reasonably high sweep scores in all of our eight populations. Compared with the non-sweep portions of the genome, this block had lower polymorphism but an increased nsyn/syn ratio (electronic supplementary material, S5), a trend similar to the one demonstrated in table 3 for sweep genes in general. Most of the nsyn polymorphisms occurred at positions where there was no variation among paralogues in the reference, indicating they were bona fide polymorphic sites as opposed to artefacts of mis-mapping onto paralogues (data not shown).

We found the linker histone (H1) to be the most polymorphic among all histone genes, consistent with the fact that it is the least conserved among histones (electronic supplementary material, S5). The basic structure of histone H1 consists of three main domains: a lysine-rich C-terminal domain that binds to linker DNA (the C domain), a central globular domain with a winged helix motif that binds to the nucleosomal DNA (the G domain), and an N-terminal domain whose function is not very well understood (the N domain) [26]. Generally, the globular domain is the most evolutionarily conserved (across taxa and among paralogues) and the N-terminal domain is the most variable [27].

Examination of polymorphic sites in H1 genes confirmed our expectations based on the known evolutionary patterns. The G and N domains showed the lowest and highest propensity for nsyn mutations, respectively (electronic supplementary material, S6, p. 2). The nsyn mutations were quite uncommon in regular secondary structures and in buried residues (electronic supplementary material, S6, pp. 3–4), although these two states tended to coincide with the globular domain, confounding the analysis. Charge-altering mutations were also exceedingly rare in buried residues (electronic supplementary material, S6, p. 7). About 90% of the changes adding or removing Ser/Thr occurred in exposed residues (electronic supplementary material, S6, p. 8) making them potential targets for epigenetic modification.

Polymorphisms converting other amino acids to Pro were vastly overrepresented in the histone block in all of our eight samples (*p* < 0.0001). We did not test for other amino acid conversions, so there may well be other cases of overrepresentation or underrepresentation in the data that we did not capture. Remarkably, the positions of Pro-permissive mutations in the conserved G domain were much more consistent across populations compared with the N domain. Among the Pro-permissive residues in the G domain, 78.6% showed Pro mutations in multiple populations. By contrast, only 17.5% of Pro-permissive residues in the N domain showed Pro mutations in more than one population (electronic supplementary material, S6, p10). This suggested that Pro mutations in the N domain were mostly neutral or semi-neutral segregating variants in random positions, whereas at least some of the Pro mutations in the G domain happened at specific positions and were probably favoured by selection. Visual inspection of the Pro polymorphisms in G and C domains suggested that almost all of them occurred in irregular parts that connected the regular secondary structures or were located on the domain boundaries.

Generally, nsyn polymorphisms and specifically those involving Pro occurred at similar residues in same-species populations, but were independently positioned when two populations of different species were compared (electronic supplementary material, S7). This verified the expectation of efficient isolation of the *C. pipiens* and *C. torrentium* gene pools. Therefore, whatever the evolutionary force behind the overabundance of conversions to Pro might be, it seems to be happening independently and in parallel in *C. pipiens* and *C. torrentium*.

## 4. Discussion

### (a) Diversity level and population structure

The three pure biotype molestus populations (A1, M1 and S1) showed reduced variation compared with the one pure biotype pipiens population (A4) (table 1) consistent with previous findings suggesting founder effects during the establishment of molestus populations [28–30]. The dependence of population structure on localities (table 2) agrees with previous reports on US populations [8,30,31] (but also see [32]) but contrasts with the distinct f. molestus versus f. pipiens dichotomy in northern and central Europe [8,29,33].

### (b) Mapping efficiency and coverage effects

Theoretical and computational tools are still being developed for pool-seq data analysis [34–36]. It has been proposed that estimates of allele frequencies and population genetic parameters can be improved with increased sequencing depth (up to 20–30×, but not above that [34,36]) and pooling large enough numbers of individuals (about 25 diploid individuals or more [36]). The recommendation of coverage threshold of 20–30× in [34,36] was made to ensure faithful estimates of ‘single-site’ allele frequencies. When diversity or selection parameters are calculated by averaging over 10 kb windows or the length of genes—which are typically several hundred bases long—even lower coverages ought to yield satisfactory results. Accordingly, in a study using simulated data of up to 2% divergence from reference sequence, it has been suggested that direct estimation of population genetic parameters without SNP and genotype calling yields reasonably good results even at low coverages (2–4×) [37]. The methods we used for estimation of diversity and selection strength worked directly on base counts from sequencing reads without any intermediate SNP or genotype calling steps. The coverage in our included genic positions ranged 8–19×, which lay between the two above recommendations. Nevertheless, the relatively low sequencing depth in some regions could have resulted in false negatives in the detection of selection targets, because they were disregarded by our 4–40× filter.

### (c) Signature of directional selection in coding sequences

It is well known that some AFS-based metrics of positive selection are sensitive to demography or behave similarly under purifying selection and positive selection (e.g. Tajima's *D*) [38]. Some false positives might exist among the Pool-hmm hits too, but we are optimistic that most of the detected sweeps are likely to be true positives. Pool-hmm identifies sweep regions based on changes in the AFS regardless of the annotatory features of the alleles; thus, the combination of lower levels of variation and higher ratios of nsyn/syn (table 3) provides independent support for the action of positive selection. Purifying selection would reduce total variation but would also decrease the nsyn/syn ratio. Relaxation of selection would increase the nsyn/syn ratio but would also elevate total variation. Still, alternative scenarios can be envisaged that would produce the kind of pattern we see in our data; for example, a severe decline in population size (a bottleneck) could result in reduced total variation and selection relaxation at the same time. The mathematical models from which the Pool-hmm method was devised were shown through simulations and tests on real data to be relatively robust to several types of demographic changes [18,19]. But unfortunately in the absence of ecological data on our *Culex* populations, the results of such simulations cannot be confidently extended to them. Further work will be required to disentangle true sweep signals from potential confounders.

A closer look at table 3 reveals that the higher nsyn/syn ratio in sweep regions resulted mainly from depletion of syn mutations. The reason is that in general most of the neutrally segregating variation is syn; so when linked variation is removed from around a sweep site, the reduction in syn variation will be larger. A possible explanation for more abundant nsyn mutations in sweep genes in M2 and M4 when no or a weaker correlation is observed for syn mutations is that a larger proportion of sweeps in *C. torrentium* were fostered by new mutations (hard sweeps), whereas most of *C. pipiens* sweeps depended on standing variation (soft sweeps). This scenario seems particularly likely in the case of the M4 population, which has very low levels of standing variation—reflected by smallest number of polymorphic sites in non-swept genes (table 3) and smallest *π* and *θ* values (table 1)—providing little raw material for positive selection. Accordingly, M4 shows the lowest number of detected sweep events (table 3). In the Moscow region, populations of *C. torrentium* have expanded rapidly in the past 10 years [39] indicating that small genetic diversity within M2 and M4 may be owing to founder effect.

An excess of nsyn to syn ‘fixed’ differences (divergence) among multiple taxa is often used as a basis for inference of recurrent positive selection [40–42]. The sites identified by those tests are likely to be the direct targets of selection and emerge owing to positive selection on the nsyn mutations. By contrast, what we present in table 3 is the ratio of nsyn/syn ‘segregating polymorphisms’ (not fixed differences) averaged over the approximately 20 k genes in individual populations, not for single-nucleotide positions across populations or taxa. What we have shown here is that an excess of nsyn/syn ‘segregating polymorphisms’ concurs with selective sweeps.

### (d) Principal component analysis on selection metrics as a method of detecting differential selection

PCA across samples on Pool-hmm scores compares the strength of recent adaptive evolution on genes, whereas PCA on Tajima's *D* captures the contrast between balancing selection (*D* > 0) and positive or purifying selection (*D* < 0) against neutral evolution (*D* = 0) [43]. In either case, the strong correlation of PC 1 with all sample labels meant that most of the genes performed similar functions and were thus selected congruently across the tested populations. This would be expected as our populations all belonged to the same species or closely related ones. On the other hand, small fractions of genes were expected to underlie adaptations unique to each specific population or groups of populations and were supposed to contribute to creation of second, third, fourth and further PCs. In the PCA on all eight samples, the second component separated *C. pipiens* samples from *C. torrentium* ones, indicating that the differential selection of genes was driven primarily by species differences (figure 1*c*). The portion of variance explained by the second component in the eight sample analysis (17.47%) was higher than that explained by any other second or third component, implying that interspecific differences were greater than those caused by geographical isolation of conspecifics.

PC patterns of sweep scores and allele frequencies share a common feature: they are shaped first by species identity and then by geographical distance. Migration between geographically close populations may have contributed to the similarity of allele frequencies and consequently, the detected targets of selection; so do the PCs of Pool-hmm merely reflect population structure? The answer is interestingly NO. First, we need to point out a key difference: in contrast with sweep scores, we do not see a PC1 correlating highly with all of the population labels with allele frequencies. The reason is that we did the PCA only on the polymorphic positions to save on computation time. The majority of genomic positions were fixed for the same allele across all populations and were filtered out. So, for allele frequencies, significant differentiation among populations starts with PC1. PC1 and PC2 of allele frequencies are qualitatively comparable to PC2 and PC3 of sweep scores. Comparing PCA of allele frequencies and Pool-hmm score shows that the order of clustering among the six *C. pipiens* populations is different between them. PC1 of allele frequencies separates Moscow and Aleksin from Sacramento (electronic supplementary material, S2). Data in table 2 confirm that the allele frequencies of the Moscow and Aleksin populations have more similarity than either do to Sacramento populations. On the contrary, PC1 and particularly PC2 of sweep scores put Aleksin populations closer to Sacramento than Moscow (figure 1). This means that within the *C. pipiens* species, sweep status does not follow population structure. Besides, *F*_st_ between collocal populations is just slightly smaller than *F*_st_ between different localities (table 2); for example, *F*_st_ between A1 and A4 is 0.166, whereas *F*_st_ between A1 and Sacramento populations is in the range of 0.160–0.193. This makes it unlikely that gene flow between collocal populations is sufficiently strong to create similar AFS in them and to lead to corresponding sweep hits. Finally, it should be noted that allele frequency PCA plots were created from biallelic positions (a fraction of polymorphic positions) regardless of gene content; so, presumably most of them came from non-genic parts because only approximately 110 MB out of the approximately 579 MB of the reference genome is genic sequence (including introns), not to mention that polymorphism is expected to be lower in genic sequences on average. On the other hand, Pool-hmm PCA plots used the sweep scores from genic sequences, consisting of monomorphic as well as polymorphic sites. So the two sets of PCA plots represent two potentially overlapping but completely different subsets of genomic positions. Without a formal significance test, it is not possible to statistically disprove or quantitatively evaluate the proposition that demography affects our ‘detected’ selective status. What we can ascertain definitely is that Pool-hmm and Tajima's *D* do not absolutely follow *F*_st_, phylogenetic neighbourhood or allele frequency PCs. In other words, the variation in neither of the former two can be completely explained by any of the latter three (contribution to the signal of selection is possible but it is never 100%).

We performed PCA on Pool-hmm scores and Tajima's *D* but it can be as effectively applied to any other selection statistics. There are a great number of indicators of natural selection (including those based on amino acid substitutions, length of haplotype homozygosity, etc.), each optimized to identify certain types of selection and within certain time depths (reviewed, for example, in [44]). PCA will make it possible to use any of them comparatively across populations or taxa to characterize the patterns of differential selection.

From an epidemiological perspective, the dependence of population structure on geographical distribution and strong local signature of adaptations suggest that vector control schemes should be informed by population-specific data rather than presumed global properties of the *Culex* species complex. For instance, rapid evolution of many insecticide resistance genes indicates that the efficacy of insecticides on each *Culex* population will have to be tested frequently and on specimens from the same locality with as small gridding as possible.

### (e) The special case of histones

Histones are known to be among the most evolutionary conserved genes, although they have been reported to have responded to recent directional selection [45,46]. However, the selective pressures that drive their evolution are not well understood.

Analysis of the biochemical versus structural properties of amino acid residues at polymorphic sites suggested that Pro mutations in the G and C domains of H1 probably acted to modify the orientation of regular structures with respect to each other in space or adjust the rigidity/flexibility of the existing structures without disrupting the basic fold of the protein or its function.

Because seasonal variation in temperature is more substantial in temperate climates compared with tropical or sub-tropical zones, sub-functionalization or neofunctionalization of duplicated genes [47] to accommodate these new environmental conditions seems like a possible scenario for *Culex* histone evolution. This scenario is corroborated by the heterogeneous distribution of polymorphic sites among paralogues in all of the studied populations (independence *χ*^2^, *p* < 0001; caution: test statistic might have been inflated owing to small number of expected polymorphisms at some loci).

### (f) The marks of south to north range expansion

*Culex pipiens* originated in North Africa and then spread out to colonize other parts of the world [48]. We found certain sweep events that might have helped them adapt to the new environmental conditions (electronic supplementary material, S4).

Heat-shock proteins and chaperonins are crucial for proper protein folding in the cells and also contribute to adaptation to living at high and low temperatures [49,50]. Interestingly, expression of a specific chaperonin component has been reported to be crucial for cold resistance in diapausing members of the Onion Maggot *Delia antiqua* [51,52]. Positive selection on chaperonins may be a general response to colder climate or bigger seasonal fluctuations in temperature; however, the significantly higher number of sweep genes in *C. pipiens* compared with *C. torrentium* makes it more likely to be a specialized adaptation to winter diapause in the colder habitats. Biotype molestus mosquitoes do not undergo diapause during winter, but they have branched off from the pipiens form very recently, so it should not be surprising that they still bear the genomic signatures of diapause-related adaptations.

Chromatin-related factors including histones showed signals of strong sweep in all of the tested populations, and constituted the most significantly enriched GO terms. As major modulators of gene expression, they are known to contribute substantially to adaptation to new environmental challenges [53,54]. Positive selection in several chromatin remodelling genes has been associated with range expansion from tropical to temperate environments in *Drosophila* [55,56]; it is then plausible to suggest that these genes may have played an equally important adaptive role during the spread of *Culex* from tropical North Africa to temperate and cold habitats. Interestingly, many regulatory sequences and unannotated sequences have been reported to be highly differentiated between tropical and temperate *Drosophila* populations, presumably contributing to adaptation to new environmental conditions [56]. Sweeps in histones and chromatin modifiers along with the large proportion of sweeps occurring in non-coding regions emphasize the significance of gene expression regulation as a mechanism of adaptive evolution in *Culex*.

## Supplementary Material

ESM

## Data accessibility

The Illumina data are available at NCBI under the BioProject PRJNA284197.
