# Supplementary material for: Evolutionary genomics of Culex pipiens: global and local adaptations associated with climate, life-history traits and anthropogenic factors
Source: Proc Biol Sci. 2015 Jul 7;282(1810):20150728. doi: 10.1098/rspb.2015.0728 (PMC4590483; doi:10.1098/rspb.2015.0728)
Supplement: ESM [file rspb20150728supp1.pdf]

# Supplementary file 1: Methods details

## Mosquito samples

Mosquito samples were taken from urban and suburban areas in Sacramento (California, US), Moscow and Aleksin (Central Russia). This strategy was based on the general supposition that urban samples would correspond to the molestus from and the suburban samples to the pipiens form of *Culex pipiens*.

*Aleksin samples.* The Aleksin urban sample (A1) consisted of mosquito larvae collected from an above-ground pond in the water-purification station located at center of Aleksin, surrounded by a small forested park. Samples were collected in August 2011. The suburban sample from Aleksin (A4) consisted of larvae collected from barrels at the garden plots in the holiday village Bogucharovo, 11 km southeast of Aleksin. The location is surrounded by forests and farmlands. There was no visible constraint except distance which could limit gene flow between natural and urban populations from Aleksin.

*Moscow samples*. The Moscow urban sample (M1) consisted of larvae taken in autumn 2011 from a laboratory culture of *Cx. p.* f. *molestus* maintained at the Biological Evolution Department of Moscow State University. Initial individuals for the culture had been caught in autumn 2008 in the basement puddle of a Moscow house. Two *C. torrentium* samples were taken from natural habitats around Moscow. M2 sample consisted of larvae and imagoes collected in July 2011 in the Semkhoz train station near Sergiev Posad situated 60 km to the north from Moscow. Larvae were taken from a wash-basin located near the forest specifically for mosquito collection. M4 were collected at MSU Zvenigorod Biological station which is situated 50 km to the west from Moscow. This station has for a century played the role of a relatively undisturbed near-Moscow territory for biological studies with many relict habitats and ecosystems. Larvae were collected in July 2011.

*Sacramento samples.* The Sacramento urban sample consisted of male specimens caught by vacuum aspiration at a utility hole in Old Sacramento in August 2011. The suburban samples were taken from catch basins at Sacramento zoo (August 2011) and Bartley Cavanaugh golf course outside the urban district (April 2011). All sites are known to support autogenous populations throughout the year.

## Sequencing, SNP calling and Annotation

*Sequencing.* Genomic DNA were prepared from mosquitoes collected for each of the eight populations into eight libraries and sequenced as paired-ended 101bp reads on an Illumina HiSeq. This process generated 407 million reads and 41 billion base pairs of nucleotide sequences. Sequenced reads were aligned as pairs using BWA 0.5.7 [1] to the complete *Culex quinquefasciatus* draft genome downloaded from the Broad Institute. Reads were allowed up to 12 mismatches throughout the 101bp per end, and unique reads were mapped to the genome. Unique reads were defined as those that mapped to only one position in the reference and were identified as having the "XT:A:U" tag. All other BWA alignment parameters were set to default values. Approximately 61% of the sequenced reads mapped uniquely to the *C. quinquefasciatus* draft genome, resulting in 42X total coverage of 461MB of the genome. Median and average coverage ranged 3-6X and 2-8X across the eight samples, respectively.

*SNP-calling and Annotation.* SNP calling was done using the GATK Unified Genotyper [2] after base quality score recalibration, indel realignment, and duplicate removal across all eight samples simultaneously [3]. We detected 6,685,360 segregating sites that contained more than one allele among the samples, of which all alleles were called either fixed in one population or existing in more than one population. Polymorphic and monomorphic sites identified by this method were subsequently used for calculation of $F_{st}$ and creation of phylogenetic trees.

## Population genetic analyses

*Species and form designation.* Reads mapping to the Barcode region of the Cytochrome Oxidase subunit I gene was used to check species identities [4,5]. The CQ11 microsatellite locus was likewise analyzed to identify the samples as pipiens or molestus form [6,7].

*Genome-wide distribution of variation.* The two polymorphism parameters $\pi$ and $\theta$ were calculated for 10kb non-overlapping sliding windows using the software Popoolation version 1.2.2 [8]. This software incorporates methods to correct for biases due to pooled sequencing in estimation of the aforementioned parameters. Only positions with coverage in the range of 4-40X were used and the minimal legitimate count for the minor allele was set to 2. Synonymous and nonsynonymous polymorphisms were assigned using the same software and the .gff file downloaded from the Broad Institute website.

*Population differentiation and admixture.* $F_{st}$ was calculated for 10kb sliding windows between each pair of populations according to the methods used in [9,10] and averaged across the genome. Maximum likelihood phylogenetic trees were constructed from sliding windows of non-overlapping 10kb using RAxML [11]. Only positions monomorphic in all samples were

included. The resulting trees were subsequently examined to find neighborhood status of samples across the sliding windows. $F_{st}$ is calculated using information from both polymorphic and monomorphic sites. Through the exclusive use of monomorphic sites in creation of the phylogenetic trees, we attempted to capture deeper differentiation events that have resulted in fixed differences among populations.

PCA on allele frequencies was also performed to examine population structure, once with all the 8 populations and once only with the 6 *C. pipiens* ones. In either case, only biallelic positions with coverage 4-40X were included in the analysis. Allele frequencies were calculated for the wild type allele at each position (not necessarily the reference allele). Individual allele frequencies at each position were centered on the mean of frequencies of that position across populations. PCA was done on these mean-centered frequencies of wild type alleles.

Divergence (proportion of "fixed" differences) of each of the populations from the reference sequence was calculated as the fraction of sites with coverage $6 \leq n \leq 35$ where at least n-1 bases were one type of derived (non-reference) base. In other words, only one base other than the major derived allele was allowed for a site to be considered fixed for the derived allele. The reason for choosing the coverage range of [6-35] was that in this interval, with one mismatch, the null hypothesis of fixation for a derived allele and sequencing error rate of 1% (typical of Illumina) would not be rejected with a one-tailed binomial test at $\alpha$=0.05.

*Genomic signatures of natural selection.* Two different methods were used to identify regions under selection. Tajima's D was obtained for genes and for 10kb non-overlapping sliding windows using Popoolation 1.2.2. The same coverage and minor allele count filter as for the calculation of pi and theta (above) was applied. Alternatively, a Hidden Markov Model-based model incorporated into the software package Pool-hmm was used to identify the selective sweep regions [12]. To parallelize the Pool-hmm process, it was run in two steps. First, allele frequency spectrum (AFS) was built based on the whole genome for each sample with the acceptable coverage range of 4-40X, theta=0.02 (based on the Popoolation output, see results) and sampling ratio of 20 (5% of positions were used for estimation of AFS). Second, sweep regions were detected separately for each supercontig (parallelized) with the same coverage range as above and transition probability of k=1e-6 based on the AFS created in the previous step.

Gene Ontology (GO) enrichment analysis on targets of selection was performed using the online software GOEAST [13]. GO annotations for *Culex* genes were downloaded from vectorbase.org/biomart. The annotation file was slightly reformatted in Python to be usable by GOEAST, and was used as the default background set. For each population two enrichment tests were run with different selected gene sets: 1) 200 genes (~1% of the total number of genes in the genome) with highest pool-hmm scores, and 2) 200 genes with lowest Tajima's D.

Enrichments with FDR<0.1 were regarded as significant. There is a body of literature on gene length bias in GO analysis of RNA-seq results [14,15]. The bias with the RNA-seq data stems from higher power for detection of differential expression in longer genes. We set out to investigate if our GO analysis on the genomic hits for selection also suffered this bias. For each population, we had done the GO analyses on the 200 genes (~1% of total gene count) with highest pool-hmm scores and 200 genes with lowest Tajima's D. So, we decided to compare the length of genes included in those selected groups with the rest of the genes. We created a flag variable indicating whether a gene belonged to the group of 200 highest pool-hmm scores in each of the 8 populations. Then, we performed a two-way ANOVA to test the association of gene length with the state of this flag (0 or 1) and the population it came from. We repeated the same test on the genes with lowest Tajima's D.

**Case study of histones**

Secondary structure prediction and calculation of solvent accessibility were done via the online Jpred server [16]. Delineation of domain boundaries was achieved through multiple sequence alignment of *Culex* histones with similar sequences from human, chicken, the midge *Chironomus pallidivittatus* and fruit fly – for which domain annotations were available on Uniprot.

As a matter of interest, we checked for overrepresented aminoacid conversions among the polymorphic positions. To avoid loss of power due to issues of multiple-comparison testing, we subsampled the dataset for initial hypothesis generation. Two H1 paralogs from two populations were selected at random and nonsynonymous substitutions were visually scanned (no statistical tests). An unusually high number of conversions to proline was the most conspicuous observation. Because of the well-known structural peculiarities of proline and its indirect role in epigenetic modification of histones [17–19], we decided to test the hypothesis of excess conversions to Pro in the main dataset formally. To do this, first we extracted the composition of the reference codons that had converted to Pro in the histone genes. Then, we subsampled the same compositions of codons 50 times from polymorphic positions elsewhere in the genome and compared the number of conversions to Pro with the number observed for the histone block by a *t* test.

**Statistical procedures**

All statistical analyses including calculation of descriptive statistics, correlation tests and principal component analysis (PCA) were done using SAS v9.3 and SAS JMP Pro 10.0.0.

**References:**


1. Li, H. & Durbin, R. 2009 Fast and accurate short read alignment with Burrows-Wheeler transform. *Bioinformatics* **25**, 1754–60. (doi:10.1093/bioinformatics/btp324)

2. McKenna, A. et al. 2010 The Genome Analysis Toolkit: a MapReduce framework for analyzing next-generation DNA sequencing data. *Genome Res.* **20**, 1297–303. (doi:10.1101/gr.107524.110)

3. DePristo, M. a et al. 2011 A framework for variation discovery and genotyping using next-generation DNA sequencing data. *Nat. Genet.* **43**, 491–8. (doi:10.1038/ng.806)

4. Hesson, J. C., Lundström, J. O., Halvarsson, P., Erixon, P. & Collado, A. 2010 A sensitive and reliable restriction enzyme assay to distinguish between the mosquitoes Culex torrentium and Culex pipiens. *Med. Vet. Entomol.* **24**, 142–9. (doi:10.1111/j.1365-2915.2010.00871.x)

5. Hebert, P. D. N., Cywinska, A., Ball, S. L. & deWaard, J. R. 2003 Biological identifications through DNA barcodes. *Proc. Biol. Sci.* **270**, 313–21. (doi:10.1098/rspb.2002.2218)

6. Bahnck, C. M. & Fonseca, D. M. 2006 Rapid assay to identify the two genetic forms of Culex (Culex) pipiens L. (Diptera: Culicidae) and hybrid populations. *Am. J. Trop. Med. Hyg.* **75**, 251–5.

7. Huang, S., Molaei, G. & Andreadis, T. G. 2008 Genetic insights into the population structure of Culex pipiens (Diptera: Culicidae) in the Northeastern United States by using microsatellite analysis. *Am. J. Trop. Med. Hyg.* **79**, 518–27.

8. Kofler, R., Orozco-terWengel, P., De Maio, N., Pandey, R. V., Nolte, V., Futschik, A., Kosiol, C. & Schlötterer, C. 2011 PoPoolation: a toolbox for population genetic analysis of next generation sequencing data from pooled individuals. *PLoS One* **6**, e15925. (doi:10.1371/journal.pone.0015925)

9. Remolina, S. C., Chang, P. L., Leips, J., Nuzhdin, S. V & Hughes, K. A. 2012 GENOMIC BASIS OF AGING AND LIFE-HISTORY EVOLUTION IN DROSOPHILA MELANOGASTER. *Evolution (N. Y).* **66**, 3390–3403. (doi:10.5061/dryad.94pv0)

10. Jalvingh, K. M., Chang, P. L., Nuzhdin, S. V & Wertheim, B. 2014 Genomic changes under rapid evolution: selection for parasitoid resistance. *Proc. Biol. Sci.* **281**, 20132303. (doi:10.1098/rspb.2013.2303)

11. Stamatakis, A. 2006 RAxML-VI-HPC: maximum likelihood-based phylogenetic analyses with thousands of taxa and mixed models. *Bioinformatics* **22**, 2688–90. (doi:10.1093/bioinformatics/btl446)

12. Boitard, S., Kofler, R., Françoise, P., Robelin, D., Schlötterer, C. & Futschik, A. 2013 Pool-hmm: a Python program for estimating the allele frequency spectrum and detecting selective sweeps from next generation sequencing of pooled samples. *Mol. Ecol. Resour.* **13**, 337–40. (doi:10.1111/1755-0998.12063)

13. Zheng, Q. & Wang, X.-J. 2008 GOEAST: a web-based software toolkit for Gene Ontology enrichment analysis. *Nucleic Acids Res.* **36**, W358–63. (doi:10.1093/nar/gkn276)

14. Young, M. D., Wakefield, M. J., Smyth, G. K. & Oshlack, A. 2010 Gene ontology analysis for RNA-seq: accounting for selection bias. *Genome Biol.* **11**, R14. (doi:10.1186/gb-2010-11-2-r14)

15. Mi, G., Di, Y., Emerson, S., Cumbie, J. S. & Chang, J. H. 2012 Length Bias Correction in Gene Ontology Enrichment Analysis Using Logistic Regression. *PLoS One* **7**. (doi:10.1371/journal.pone.0046128)

16. Cole, C., Barber, J. D. & Barton, G. J. 2008 The Jpred 3 secondary structure prediction server. *Nucleic Acids Res.* **36**, W197–201. (doi:10.1093/nar/gkn238)

17. Raghuram, N., Strickfaden, H., McDonald, D., Williams, K., Fang, H., Mizzen, C., Hayes, J. J., Th’ng, J. & Hendzel, M. J. 2013 Pin1 promotes histone H1 dephosphorylation and stabilizes its binding to chromatin. *J. Cell Biol.* **203**, 57–71. (doi:10.1083/jcb.201305159)

18. Nelson, C. J., Santos-Rosa, H. & Kouzarides, T. 2006 Proline isomerization of histone H3 regulates lysine methylation and gene expression. *Cell* **126**, 905–16. (doi:10.1016/j.cell.2006.07.026)

19. Harshman, S. W., Young, N. L., Parthun, M. R. & Freitas, M. A. 2013 H1 histones: current perspectives and challenges. *Nucleic Acids Res.* , 1–17. (doi:10.1093/nar/gkt700)

# Supplementary file 2: PCA of allele frequencies

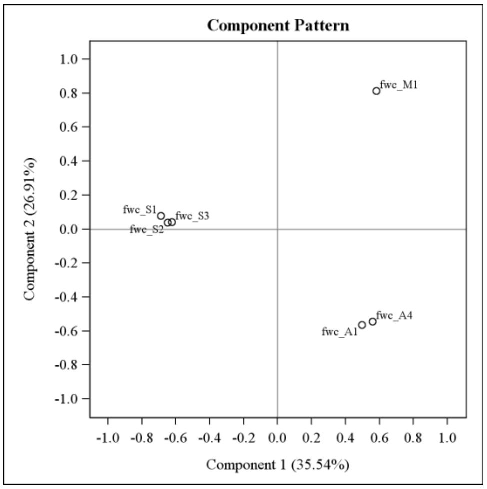


a

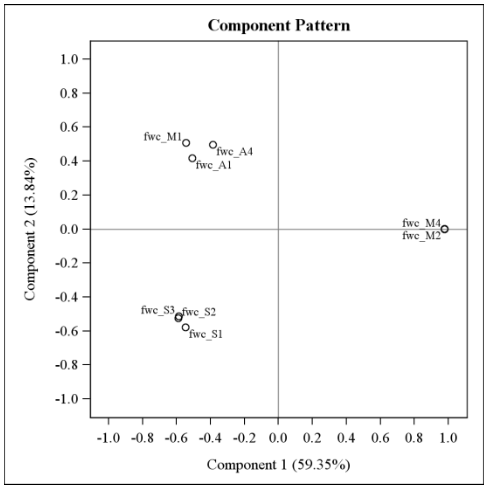


b

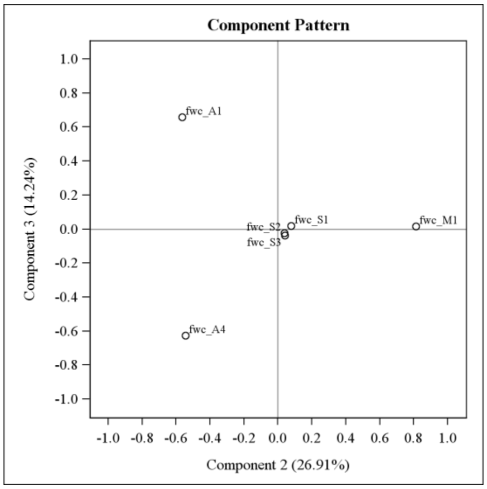


c

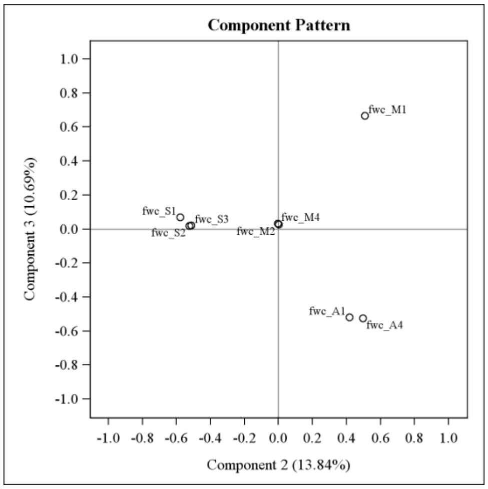


d

**Figure Suppl. 2. PCA results on allele frequencies at biallelic positions within the six *C. pipiens* populations (a,c) and the eight *Culex* populations (b,d). The "fwc" prefix stands for frequency of wild type allele, centered on the mean. Satisfying the conditions of being biallelic and having 4-40X coverage in all of the tested populations, 1'890'722 positions were used for (a,c) and 814'469 positions were used for (b,d).**

# Supplementary file 3: Summary of Tajima’s D and Pool-hmm

## Histograms and average Tajima’s D values for the 8 populations

| A1; average=-0.1896 | A4; average=-0.1650 | |
|---|---|---|
| 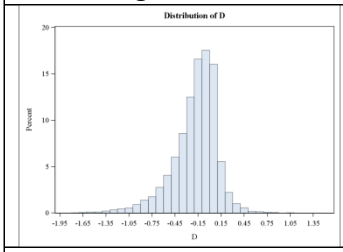  | 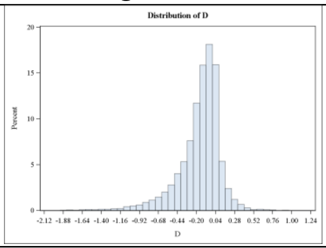  | |
| **M1; average=-0.2576** | **M2; average=-0.4509** | **M4; average=-0.0873** |
| 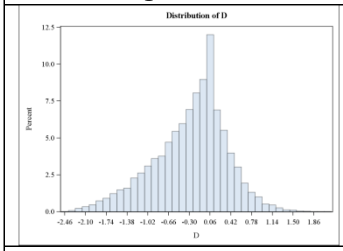  | 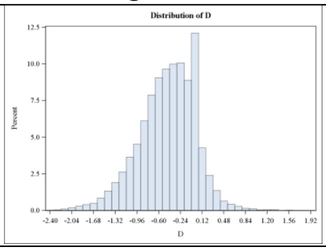  | 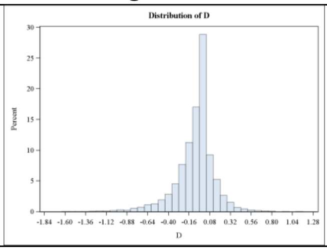  |
| **S1; average=-0.0744** | **S2; average=-0.2083** | **S3; average=-0.2129** |
| 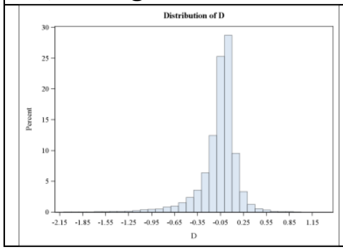  | 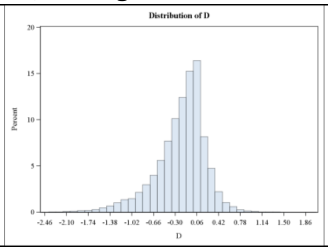  | 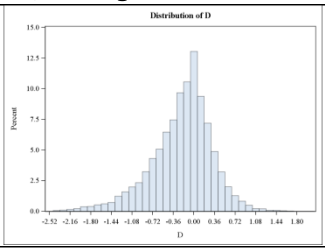  |

## Distribution of lengths and scores of the hits detected by Pool-hmm

Distributions

Score | Length

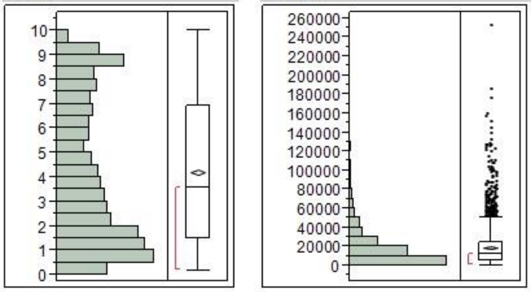


**Quantiles (Score)**

| | | |
|---|---|---|
| 100.0% | maximum | 9.98974 |
| 99.5% | | 9.68776 |
| 97.5% | | 9.33175 |
| 90.0% | | 8.72753 |
| 75.0% | quartile | 6.9319 |
| 50.0% | median | 3.56617 |
| 25.0% | quartile | 1.51579 |
| 10.0% | | 0.72327 |
| 2.5% | | 0.31833 |
| 0.5% | | 0.23289 |
| 0.0% | minimum | 0.14616 |

**Summary Statistics (Score)**

| | |
|---|---|
| Mean | 4.2114024 |
| Std Dev | 2.9590039 |
| Std Err Mean | 0.062858 |
| Upper 95% Mean | 4.3346692 |
| Lower 95% Mean | 4.0881356 |
| N | 2216 |

**Quantiles (Length)**

| | | |
|---|---|---|
| 100.0% | maximum | 252812 |
| 99.5% | | 127293 |
| 97.5% | | 80054.1 |
| 90.0% | | 44346.1 |
| 75.0% | quartile | 23932 |
| 50.0% | median | 11859 |
| 25.0% | quartile | 5789 |
| 10.0% | | 2798.5 |
| 2.5% | | 1037.43 |
| 0.5% | | 112.68 |
| 0.0% | minimum | 46 |

**Summary Statistics (Length)**

| | |
|---|---|
| Mean | 19092.483 |
| Std Dev | 21932.7 |
| Std Err Mean | 465.91556 |
| Upper 95% Mean | 20006.16 |
| Lower 95% Mean | 18178.806 |
| N | 2216 |

A1

Distributions

Score | Length

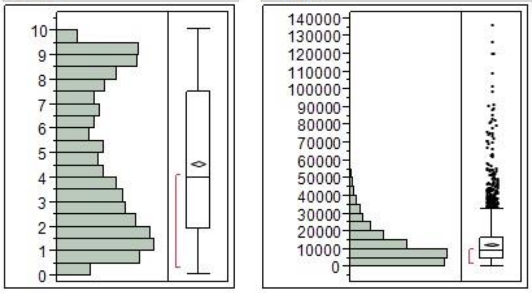


**Quantiles (Score)**

| | | |
|---|---|---|
| 100.0% | maximum | 10.0507 |
| 99.5% | | 9.66691 |
| 97.5% | | 9.42034 |
| 90.0% | | 8.93758 |
| 75.0% | quartile | 7.5329 |
| 50.0% | median | 3.98406 |
| 25.0% | quartile | 1.91062 |
| 10.0% | | 0.98947 |
| 2.5% | | 0.46275 |
| 0.5% | | 0.2306 |
| 0.0% | minimum | 0.05167 |

**Summary Statistics (Score)**

| | |
|---|---|
| Mean | 4.5934742 |
| Std Dev | 2.964949 |
| Std Err Mean | 0.0547938 |
| Upper 95% Mean | 4.7009126 |
| Lower 95% Mean | 4.4860358 |
| N | 2928 |

**Quantiles (Length)**

| | | |
|---|---|---|
| 100.0% | maximum | 135906 |
| 99.5% | | 81830.2 |
| 97.5% | | 45763.4 |
| 90.0% | | 27437.9 |
| 75.0% | quartile | 16143.8 |
| 50.0% | median | 8849.5 |
| 25.0% | quartile | 4692 |
| 10.0% | | 2634.8 |
| 2.5% | | 1285.7 |
| 0.5% | | 527.45 |
| 0.0% | minimum | 21 |

**Summary Statistics (Length)**

| | |
|---|---|
| Mean | 12695.88 |
| Std Dev | 12858.756 |
| Std Err Mean | 237.63664 |
| Upper 95% Mean | 13161.832 |
| Lower 95% Mean | 12229.928 |
| N | 2928 |

A4

Distributions

Score | Length

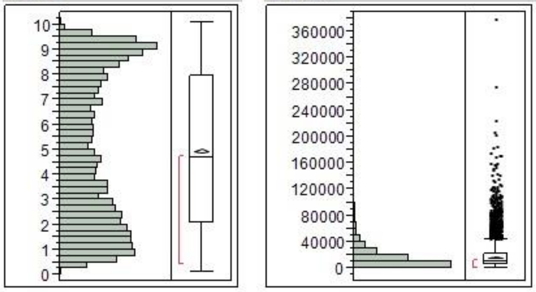


**Quantiles (Score)**

| | | |
|---|---|---|
| 100.0% | maximum | 10.0867 |
| 99.5% | | 9.69296 |
| 97.5% | | 9.46889 |
| 90.0% | | 9.05944 |
| 75.0% | quartile | 7.94763 |
| 50.0% | median | 4.66729 |
| 25.0% | quartile | 2.10511 |
| 10.0% | | 1.11016 |
| 2.5% | | 0.60769 |
| 0.5% | | 0.39517 |
| 0.0% | minimum | 0.10105 |

**Summary Statistics (Score)**

| | |
|---|---|
| Mean | 4.9532963 |
| Std Dev | 2.9972788 |
| Std Err Mean | 0.0356819 |
| Upper 95% Mean | 5.0232435 |
| Lower 95% Mean | 4.883349 |
| N | 7056 |

**Quantiles (Length)**

| | | |
|---|---|---|
| 100.0% | maximum | 377878 |
| 99.5% | | 117473 |
| 97.5% | | 71516.7 |
| 90.0% | | 37811.3 |
| 75.0% | quartile | 21089.8 |
| 50.0% | median | 10766 |
| 25.0% | quartile | 5403.25 |
| 10.0% | | 2746.4 |
| 2.5% | | 1228.85 |
| 0.5% | | 503.99 |
| 0.0% | minimum | 68 |

**Summary Statistics (Length)**

| | |
|---|---|
| Mean | 17040.626 |
| Std Dev | 19991.695 |
| Std Err Mean | 237.99637 |
| Upper 95% Mean | 17507.17 |
| Lower 95% Mean | 16574.081 |
| N | 7056 |

Distributions

Score | Length

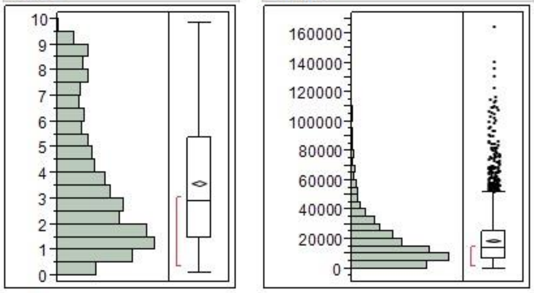


**Quantiles (Score)**

| | | |
|---|---|---|
| 100.0% | maximum | 9.85615 |
| 99.5% | | 9.32113 |
| 97.5% | | 8.96679 |
| 90.0% | | 7.86677 |
| 75.0% | quartile | 5.36524 |
| 50.0% | median | 2.8795 |
| 25.0% | quartile | 1.48314 |
| 10.0% | | 0.80589 |
| 2.5% | | 0.37118 |
| 0.5% | | 0.22458 |
| 0.0% | minimum | 0.09214 |

**Summary Statistics (Score)**

| | |
|---|---|
| Mean | 3.6052252 |
| Std Dev | 2.5644837 |
| Std Err Mean | 0.0483349 |
| Upper 95% Mean | 3.7000006 |
| Lower 95% Mean | 3.5104498 |
| N | 2815 |

**Quantiles (Length)**

| | | |
|---|---|---|
| 100.0% | maximum | 164391 |
| 99.5% | | 107366 |
| 97.5% | | 70466.4 |
| 90.0% | | 40615.8 |
| 75.0% | quartile | 24969 |
| 50.0% | median | 13373 |
| 25.0% | quartile | 6892 |
| 10.0% | | 3423.4 |
| 2.5% | | 1420.8 |
| 0.5% | | 196.64 |
| 0.0% | minimum | 58 |

**Summary Statistics (Length)**

| | |
|---|---|
| Mean | 19101.002 |
| Std Dev | 18393.242 |
| Std Err Mean | 346.67226 |
| Upper 95% Mean | 19780.76 |
| Lower 95% Mean | 18421.245 |
| N | 2815 |

M1

**Distributions**

**Score** | **Length**

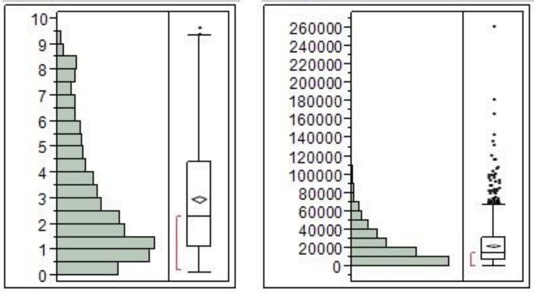


**Quantiles** (Score)

| | | |
|---|---|---|
| 100.0% | maximum | 9.65681 |
| 99.5% | | 9.10979 |
| 97.5% | | 8.22527 |
| 90.0% | | 6.8531 |
| 75.0% | quartile | 4.42915 |
| 50.0% | median | 2.26839 |
| 25.0% | quartile | 1.12052 |
| 10.0% | | 0.54818 |
| 2.5% | | 0.29339 |
| 0.5% | | 0.22056 |
| 0.0% | minimum | 0.12621 |

**Summary Statistics** (Score)

| | |
|---|---|
| Mean | 2.9767471 |
| Std Dev | 2.3337607 |
| Std Err Mean | 0.067996 |
| Upper 95% Mean | 3.1101541 |
| Lower 95% Mean | 2.8433401 |
| N | 1178 |

**Quantiles** (Length)

| | | |
|---|---|---|
| 100.0% | maximum | 261270 |
| 99.5% | | 132272 |
| 97.5% | | 82439.5 |
| 90.0% | | 51654.8 |
| 75.0% | quartile | 31099.3 |
| 50.0% | median | 14900 |
| 25.0% | quartile | 6851 |
| 10.0% | | 3035.6 |
| 2.5% | | 383.975 |
| 0.5% | | 89.895 |
| 0.0% | minimum | 20 |

**Summary Statistics** (Length)

| | |
|---|---|
| Mean | 22543.203 |
| Std Dev | 23250.191 |
| Std Err Mean | 677.41356 |
| Upper 95% Mean | 23872.276 |
| Lower 95% Mean | 21214.13 |
| N | 1178 |

M4

M2

**Distributions**

**Score** | **Length**

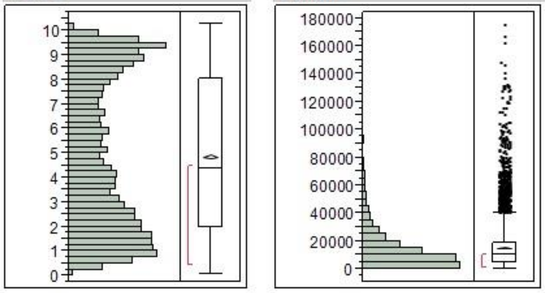


**Quantiles** (Score)

| | | |
|---|---|---|
| 100.0% | maximum | 10.26 |
| 99.5% | | 9.91397 |
| 97.5% | | 9.68887 |
| 90.0% | | 9.24483 |
| 75.0% | quartile | 8.02748 |
| 50.0% | median | 4.36092 |
| 25.0% | quartile | 2.00118 |
| 10.0% | | 1.04585 |
| 2.5% | | 0.5568 |
| 0.5% | | 0.33665 |
| 0.0% | minimum | 0.0914 |

**Summary Statistics** (Score)

| | |
|---|---|
| Mean | 4.866287 |
| Std Dev | 3.0785638 |
| Std Err Mean | 0.0371884 |
| Upper 95% Mean | 4.9391879 |
| Lower 95% Mean | 4.7933862 |
| N | 6853 |

**Quantiles** (Length)

| | | |
|---|---|---|
| 100.0% | maximum | 175407 |
| 99.5% | | 102697 |
| 97.5% | | 63267.7 |
| 90.0% | | 34657.6 |
| 75.0% | quartile | 19046.5 |
| 50.0% | median | 9820 |
| 25.0% | quartile | 4824 |
| 10.0% | | 2466.4 |
| 2.5% | | 1198.35 |
| 0.5% | | 563.89 |
| 0.0% | minimum | 53 |

**Summary Statistics** (Length)

| | |
|---|---|
| Mean | 15357.682 |
| Std Dev | 17166.818 |
| Std Err Mean | 207.37168 |
| Upper 95% Mean | 15764.195 |
| Lower 95% Mean | 14951.169 |
| N | 6853 |

S1

**Distributions**

**Score** | **Length**

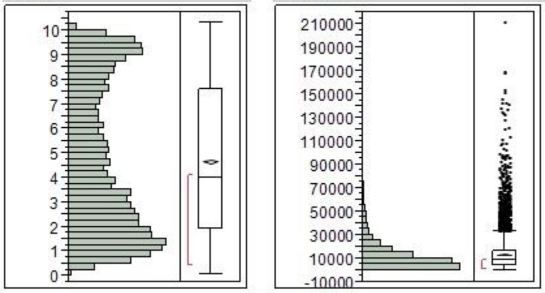


**Quantiles** (Score)

| | | |
|---|---|---|
| 100.0% | maximum | 10.3538 |
| 99.5% | | 9.96769 |
| 97.5% | | 9.72555 |
| 90.0% | | 9.18645 |
| 75.0% | quartile | 7.64107 |
| 50.0% | median | 4.00365 |
| 25.0% | quartile | 1.92101 |
| 10.0% | | 1.07797 |
| 2.5% | | 0.61047 |
| 0.5% | | 0.35093 |
| 0.0% | minimum | 0.06208 |

**Summary Statistics** (Score)

| | |
|---|---|
| Mean | 4.6829403 |
| Std Dev | 3.0275828 |
| Std Err Mean | 0.0378566 |
| Upper 95% Mean | 4.7571519 |
| Lower 95% Mean | 4.6087286 |
| N | 6396 |

**Quantiles** (Length)

| | | |
|---|---|---|
| 100.0% | maximum | 211318 |
| 99.5% | | 96736.3 |
| 97.5% | | 56446.5 |
| 90.0% | | 28865.9 |
| 75.0% | quartile | 15947.3 |
| 50.0% | median | 8454 |
| 25.0% | quartile | 4333.75 |
| 10.0% | | 2503.1 |
| 2.5% | | 1203.85 |
| 0.5% | | 623.73 |
| 0.0% | minimum | 73 |

**Summary Statistics** (Length)

| | |
|---|---|
| Mean | 13346.386 |
| Std Dev | 15617.845 |
| Std Err Mean | 195.2841 |
| Upper 95% Mean | 13729.208 |
| Lower 95% Mean | 12963.564 |
| N | 6396 |

S2

**Distributions**

**Score** | **Length**

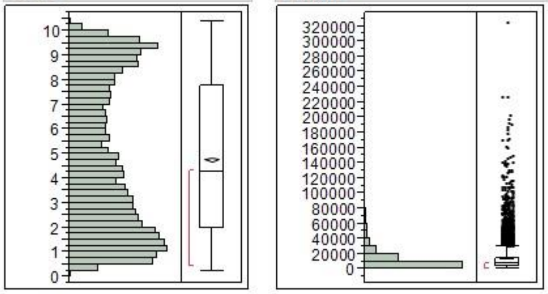


**Quantiles** (Score)

| | | |
|---|---|---|
| 100.0% | maximum | 10.3755 |
| 99.5% | | 10.0502 |
| 97.5% | | 9.73745 |
| 90.0% | | 9.22038 |
| 75.0% | quartile | 7.75163 |
| 50.0% | median | 4.25571 |
| 25.0% | quartile | 1.98237 |
| 10.0% | | 1.06425 |
| 2.5% | | 0.57979 |
| 0.5% | | 0.40613 |
| 0.0% | minimum | 0.22694 |

**Summary Statistics** (Score)

| | |
|---|---|
| Mean | 4.783812 |
| Std Dev | 3.0319618 |
| Std Err Mean | 0.0344075 |
| Upper 95% Mean | 4.8512599 |
| Lower 95% Mean | 4.716364 |
| N | 7765 |

**Quantiles** (Length)

| | | |
|---|---|---|
| 100.0% | maximum | 323685 |
| 99.5% | | 116707 |
| 97.5% | | 62521.3 |
| 90.0% | | 28302.8 |
| 75.0% | quartile | 14622.5 |
| 50.0% | median | 7495 |
| 25.0% | quartile | 3889 |
| 10.0% | | 2052.6 |
| 2.5% | | 1010.15 |
| 0.5% | | 407.66 |
| 0.0% | minimum | 181 |

**Summary Statistics** (Length)

| | |
|---|---|
| Mean | 13087.046 |
| Std Dev | 18187.446 |
| Std Err Mean | 206.39587 |
| Upper 95% Mean | 13491.637 |
| Lower 95% Mean | 12682.454 |
| N | 7765 |

S3

# Supplementary file 4: Functional analysis of sweep genes

## Gene Ontology (GO) enrichment analysis

For each population, 200 genes (~1% of the number of annotated genes in the genome) with the highest Pool-hmm scores or the most negative Tajima's D values were analyzed for enrichment of GO terms. Only terms with False Discovery Rate (FDR)<0.1 are listed.

The ANOVA test for the association of gene length with Pool-hmm was significant, but the effect was extremely small ($p=0.0002$, $R^2=0.000256$). A very similar result was obtained with the genes having the lowest Tajima's D ($p<0.0001$, $R^2=0.000899$). These tests clearly proved that gene length had not been a substantial confounder in our GO analyses on selection targets detected by either Pool-hmm or Tajima's D method. Details of how the ANOVA test was performed is provided in Suppl. 1.

**GO enrichment of top 200 Pool-hmm hits for each population:**

| | | | | |
|---|---|---|---|---|
| **A1** | | | | |
| GOID | Ontology | Term | Log odds-ratio | FDR |
| | | | | |
| **A4** | | | | |
| GOID | Ontology | Term | Log odds-ratio | FDR |
| GO:0000785 | cellular_component | chromatin | 5.299 | 6.14E-99 |
| GO:0034728 | biological_process | nucleosome organization | 5.299 | 6.14E-99 |
| GO:0071824 | biological_process | protein-DNA complex subunit organization | 5.299 | 6.14E-99 |
| GO:0006325 | biological_process | chromatin organization | 4.685 | 3.98E-84 |
| GO:0044427 | cellular_component | chromosomal part | 4.575 | 1.86E-81 |
| GO:0005694 | cellular_component | chromosome | 4.426 | 4.78E-79 |
| GO:0051276 | biological_process | chromosome organization | 4.207 | 6.72E-75 |
| GO:0043933 | biological_process | macromolecular complex subunit organization | 4.095 | 4.15E-71 |
| GO:0006996 | biological_process | organelle organization | 2.977 | 4.83E-51 |
| GO:0043228 | cellular_component | non-membrane-bounded organelle | 2.778 | 2.09E-46 |
| GO:0043232 | cellular_component | intracellular non-membrane-bounded organelle | 2.778 | 2.09E-46 |
| GO:0044446 | cellular_component | intracellular organelle part | 2.442 | 1.80E-42 |
| GO:0044422 | cellular_component | organelle part | 2.388 | 8.46E-42 |
| GO:0071840 | biological_process | cellular component organization or biogenesis | 2.395 | 1.64E-39 |
| GO:0016043 | biological_process | cellular component organization | 2.439 | 2.44E-39 |
| GO:0003676 | molecular_function | nucleic acid binding | 1.993 | 2.11E-28 |
| GO:0043231 | cellular_component | intracellular membrane-bounded | 1.51 | 3.16E-23 |

| | | | | |
|---|---|---|---|---|
| | | organelle | | |
| GO:0043227 | cellular_component | membrane-bounded organelle | 1.476 | 7.70E-23 |
| GO:0005622 | cellular_component | intracellular | 1.119 | 5.24E-22 |
| GO:0044424 | cellular_component | intracellular part | 1.128 | 6.73E-22 |
| GO:0043229 | cellular_component | intracellular organelle | 1.314 | 1.54E-21 |
| GO:0044763 | biological_process | single-organism cellular process | 1.418 | 3.16E-21 |
| GO:0043226 | cellular_component | organelle | 1.299 | 3.32E-21 |
| GO:1901363 | molecular_function | heterocyclic compound binding | 1.325 | 1.80E-19 |
| GO:0097159 | molecular_function | organic cyclic compound binding | 1.319 | 2.26E-19 |
| GO:0044699 | biological_process | single-organism process | 1.107 | 1.56E-15 |
| GO:0044444 | cellular_component | cytoplasmic part | 1.935 | 4.66E-14 |
| GO:0005623 | cellular_component | cell | 0.776 | 1.01E-13 |
| GO:0044464 | cellular_component | cell part | 0.776 | 1.01E-13 |
| GO:0005737 | cellular_component | cytoplasm | 1.893 | 1.28E-13 |
| GO:0009987 | biological_process | cellular process | 0.806 | 8.51E-13 |
| GO:0043189 | cellular_component | H4/H2A histone acetyltransferase complex | 5.379 | 1.55E-08 |
| GO:0005575 | cellular_component | cellular_component | 0.498 | 6.74E-08 |
| GO:0005488 | molecular_function | binding | 0.456 | 1.58E-07 |
| GO:0000123 | cellular_component | histone acetyltransferase complex | 4.478 | 2.31E-06 |
| GO:0008150 | biological_process | biological_process | 0.302 | 4.87E-04 |
| GO:0044428 | cellular_component | nuclear part | 1.428 | 5.47E-02 |
| GO:0005654 | cellular_component | nucleoplasm | 2.241 | 7.78E-02 |
| GO:0044451 | cellular_component | nucleoplasm part | 2.241 | 7.78E-02 |
| GO:0005634 | cellular_component | nucleus | 1.357 | 8.39E-02 |
| | | | | |
| **M1** | | | | |
| GOID | Ontology | Term | Log odds-ratio | FDR |
| | | | | |
| **M2** | | | | |
| GOID | Ontology | Term | Log odds-ratio | FDR |
| | | | | |
| **M4** | | | | |
| GOID | Ontology | Term | Log odds-ratio | FDR |
| GO:0000785 | cellular_component | chromatin | 5.362 | 3.82E-99 |
| GO:0034728 | biological_process | nucleosome organization | 5.362 | 3.82E-99 |
| GO:0071824 | biological_process | protein-DNA complex subunit organization | 5.362 | 3.82E-99 |
| GO:0006325 | biological_process | chromatin organization | 4.768 | 1.74E-86 |
| GO:0044427 | cellular_component | chromosomal part | 4.638 | 5.20E-82 |
| GO:0005694 | cellular_component | chromosome | 4.488 | 1.08E-79 |
| GO:0051276 | biological_process | chromosome organization | 4.27 | 1.17E-75 |
| GO:0043933 | biological_process | macromolecular complex subunit organization | 4.118 | 1.15E-68 |

| | | | | |
|---|---|---|---|---|
| GO:0006996 | biological_process | organelle organization | 2.987 | 1.14E-48 |
| GO:0043228 | cellular_component | non-membrane-bounded organelle | 2.768 | 3.90E-43 |
| GO:0043232 | cellular_component | intracellular non-membrane-bounded organelle | 2.768 | 3.90E-43 |
| GO:0016043 | biological_process | cellular component organization | 2.486 | 1.33E-39 |
| GO:0071840 | biological_process | cellular component organization or biogenesis | 2.425 | 6.88E-39 |
| GO:0044446 | cellular_component | intracellular organelle part | 2.386 | 8.34E-37 |
| GO:0044422 | cellular_component | organelle part | 2.333 | 2.87E-36 |
| GO:0003676 | molecular_function | nucleic acid binding | 2.146 | 2.24E-34 |
| GO:0043227 | cellular_component | membrane-bounded organelle | 1.604 | 6.86E-28 |
| GO:0043231 | cellular_component | intracellular membrane-bounded organelle | 1.623 | 1.73E-27 |
| GO:0044763 | biological_process | single-organism cellular process | 1.547 | 4.64E-26 |
| GO:0043229 | cellular_component | intracellular organelle | 1.368 | 7.09E-23 |
| GO:0043226 | cellular_component | organelle | 1.353 | 1.60E-22 |
| GO:0044699 | biological_process | single-organism process | 1.234 | 5.60E-20 |
| GO:1901363 | molecular_function | heterocyclic compound binding | 1.36 | 7.91E-20 |
| GO:0097159 | molecular_function | organic cyclic compound binding | 1.355 | 9.87E-20 |
| GO:0044444 | cellular_component | cytoplasmic part | 2.107 | 2.03E-17 |
| GO:0005737 | cellular_component | cytoplasm | 2.065 | 6.69E-17 |
| GO:0044424 | cellular_component | intracellular part | 1.039 | 2.70E-16 |
| GO:0005622 | cellular_component | intracellular | 1.018 | 8.13E-16 |
| GO:0009987 | biological_process | cellular process | 0.81 | 3.48E-12 |
| GO:0005623 | cellular_component | cell | 0.707 | 5.06E-10 |
| GO:0044464 | cellular_component | cell part | 0.707 | 5.06E-10 |
| GO:0043189 | cellular_component | H4/H2A histone acetyltransferase complex | 5.461 | 1.08E-08 |
| GO:0000123 | cellular_component | histone acetyltransferase complex | 4.561 | 1.71E-06 |
| GO:0005488 | molecular_function | binding | 0.437 | 2.86E-06 |
| GO:0005575 | cellular_component | cellular_component | 0.451 | 1.10E-05 |
| GO:0016863 | molecular_function | intramolecular oxidoreductase activity, transposing C=C bonds | 5.824 | 1.40E-03 |
| GO:0006570 | biological_process | tyrosine metabolic process | 5.146 | 7.47E-03 |
| GO:0008150 | biological_process | biological_process | 0.262 | 1.56E-02 |
| GO:0009072 | biological_process | aromatic amino acid family metabolic process | 4.561 | 2.70E-02 |
| GO:0016860 | molecular_function | intramolecular oxidoreductase activity | 4.239 | 5.06E-02 |
| GO:0005654 | cellular_component | nucleoplasm | 2.324 | 5.09E-02 |
| GO:0044451 | cellular_component | nucleoplasm part | 2.324 | 5.09E-02 |
| | | | | |
| **S1** | | | | |
| GOID | Ontology | Term | Log odds-ratio | FDR |
| GO:0000785 | cellular_component | chromatin | 5.262 | 1.71E-96 |

| | | | | |
|---|---|---|---|---|
| GO:0034728 | biological_process | nucleosome organization | 5.262 | 1.71E-96 |
| GO:0071824 | biological_process | protein-DNA complex subunit organization | 5.262 | 1.71E-96 |
| GO:0006325 | biological_process | chromatin organization | 4.647 | 5.79E-82 |
| GO:0044427 | cellular_component | chromosomal part | 4.518 | 1.31E-77 |
| GO:0005694 | cellular_component | chromosome | 4.348 | 1.22E-73 |
| GO:0051276 | biological_process | chromosome organization | 4.131 | 9.95E-70 |
| GO:0043933 | biological_process | macromolecular complex subunit organization | 4.058 | 3.56E-69 |
| GO:0006996 | biological_process | organelle organization | 2.868 | 8.67E-45 |
| GO:0043228 | cellular_component | non-membrane-bounded organelle | 2.724 | 8.98E-44 |
| GO:0043232 | cellular_component | intracellular non-membrane-bounded organelle | 2.724 | 8.98E-44 |
| GO:0016043 | biological_process | cellular component organization | 2.331 | 6.40E-34 |
| GO:0003676 | molecular_function | nucleic acid binding | 2.063 | 2.46E-32 |
| GO:0071840 | biological_process | cellular component organization or biogenesis | 2.252 | 2.56E-32 |
| GO:0044422 | cellular_component | organelle part | 2.196 | 1.04E-31 |
| GO:0044446 | cellular_component | intracellular organelle part | 2.23 | 2.18E-31 |
| GO:1901363 | molecular_function | heterocyclic compound binding | 1.352 | 5.82E-21 |
| GO:0097159 | molecular_function | organic cyclic compound binding | 1.347 | 7.27E-21 |
| GO:0044763 | biological_process | single-organism cellular process | 1.304 | 8.37E-17 |
| GO:0043227 | cellular_component | membrane-bounded organelle | 1.279 | 3.36E-15 |
| GO:0043231 | cellular_component | intracellular membrane-bounded organelle | 1.293 | 6.08E-15 |
| GO:0043229 | cellular_component | intracellular organelle | 1.114 | 1.38E-13 |
| GO:0043226 | cellular_component | organelle | 1.099 | 2.60E-13 |
| GO:0044444 | cellular_component | cytoplasmic part | 1.886 | 3.33E-13 |
| GO:0005737 | cellular_component | cytoplasm | 1.845 | 9.36E-13 |
| GO:0044699 | biological_process | single-organism process | 0.998 | 7.77E-12 |
| GO:0005622 | cellular_component | intracellular | 0.861 | 9.25E-11 |
| GO:0044424 | cellular_component | intracellular part | 0.867 | 1.06E-10 |
| GO:0043189 | cellular_component | H4/H2A histone acetyltransferase complex | 5.361 | 1.38E-08 |
| GO:0009987 | biological_process | cellular process | 0.656 | 1.58E-07 |
| GO:0000123 | cellular_component | histone acetyltransferase complex | 4.461 | 2.10E-06 |
| GO:0005488 | molecular_function | binding | 0.416 | 4.40E-06 |
| GO:0005623 | cellular_component | cell | 0.539 | 2.15E-05 |
| GO:0044464 | cellular_component | cell part | 0.539 | 2.15E-05 |
| GO:0005654 | cellular_component | nucleoplasm | 2.416 | 1.13E-02 |
| GO:0044451 | cellular_component | nucleoplasm part | 2.416 | 1.13E-02 |
| | | | | |
| **S2** | | | | |
| GOID | Ontology | Term | Log odds-ratio | FDR |
| | | | | |

| **S3** | | | | |
|---|---|---|---|---|
| GOID | Ontology | Term | Log odds-ratio | FDR |
| GO:0032991 | cellular_component | macromolecular complex | 1.062 | 5.32E-02 |
| GO:0000166 | molecular_function | nucleotide binding | 0.935 | 5.37E-02 |
| GO:1901265 | molecular_function | nucleoside phosphate binding | 0.935 | 5.37E-02 |
| GO:0036094 | molecular_function | small molecule binding | 0.913 | 5.66E-02 |
| GO:0005622 | cellular_component | intracellular | 0.54 | 6.52E-02 |
| GO:0030529 | cellular_component | ribonucleoprotein complex | 1.402 | 6.52E-02 |
| GO:0012505 | cellular_component | endomembrane system | 2.301 | 6.52E-02 |

**GO enrichment of 200 genes with the most negative Tajima's D for each population:**

| **A1** | | | | |
|---|---|---|---|---|
| GOID | Ontology | Term | Log odds-ratio | FDR |
| GO:0000785 | cellular_component | chromatin | 2.941 | 4.55E-05 |
| GO:0006996 | biological_process | organelle organization | 1.655 | 4.55E-05 |
| GO:0051276 | biological_process | chromosome organization | 2.259 | 7.57E-05 |
| GO:0034728 | biological_process | nucleosome organization | 2.825 | 1.24E-04 |
| GO:0043933 | biological_process | macromolecular complex subunit organization | 2.167 | 1.24E-04 |
| GO:0071824 | biological_process | protein-DNA complex subunit organization | 2.825 | 1.24E-04 |
| GO:0044427 | cellular_component | chromosomal part | 2.403 | 1.31E-04 |
| GO:0043228 | cellular_component | non-membrane-bounded organelle | 1.456 | 1.92E-04 |
| GO:0043232 | cellular_component | intracellular non-membrane-bounded organelle | 1.456 | 1.92E-04 |
| GO:0016043 | biological_process | cellular component organization | 1.322 | 1.92E-04 |
| GO:0005694 | cellular_component | chromosome | 2.234 | 3.76E-04 |
| GO:0071840 | biological_process | cellular component organization or biogenesis | 1.243 | 4.73E-04 |
| GO:0044424 | cellular_component | intracellular part | 0.648 | 7.97E-04 |
| GO:0005622 | cellular_component | intracellular | 0.627 | 1.36E-03 |
| GO:0006325 | biological_process | chromatin organization | 2.191 | 5.00E-03 |
| GO:0032991 | cellular_component | macromolecular complex | 1.053 | 5.00E-03 |
| GO:0044422 | cellular_component | organelle part | 1.039 | 1.91E-02 |
| GO:0007049 | biological_process | cell cycle | 1.638 | 3.15E-02 |
| GO:0044446 | cellular_component | intracellular organelle part | 1.017 | 4.04E-02 |
| GO:0022402 | biological_process | cell cycle process | 1.735 | 7.84E-02 |
| | | | | |
| **A4** | | | | |
| GOID | Ontology | Term | Log odds-ratio | FDR |
| GO:0000785 | cellular_component | chromatin | 4.648 | 1.12E-45 |
| GO:0034728 | biological_process | nucleosome organization | 4.614 | 1.71E-44 |

| | | | | |
|---|---|---|---|---|
| GO:0071824 | biological_process | protein-DNA complex subunit organization | 4.614 | 1.71E-44 |
| GO:0044427 | cellular_component | chromosomal part | 3.904 | 5.65E-36 |
| GO:0006325 | biological_process | chromatin organization | 3.979 | 5.65E-36 |
| GO:0005694 | cellular_component | chromosome | 3.767 | 3.96E-35 |
| GO:0051276 | biological_process | chromosome organization | 3.562 | 2.89E-33 |
| GO:0043933 | biological_process | macromolecular complex subunit organization | 3.437 | 7.78E-31 |
| GO:0006996 | biological_process | organelle organization | 2.344 | 1.19E-19 |
| GO:0003676 | molecular_function | nucleic acid binding | 1.785 | 2.38E-18 |
| GO:0071840 | biological_process | cellular component organization or biogenesis | 1.927 | 1.69E-17 |
| GO:0016043 | biological_process | cellular component organization | 1.955 | 3.85E-17 |
| GO:0043228 | cellular_component | non-membrane-bounded organelle | 2.115 | 1.80E-16 |
| GO:0043232 | cellular_component | intracellular non-membrane-bounded organelle | 2.115 | 1.80E-16 |
| GO:0044446 | cellular_component | intracellular organelle part | 1.873 | 4.23E-16 |
| GO:0044422 | cellular_component | organelle part | 1.802 | 3.70E-15 |
| GO:0044424 | cellular_component | intracellular part | 0.885 | 3.31E-10 |
| GO:0097159 | molecular_function | organic cyclic compound binding | 1.086 | 3.31E-10 |
| GO:1901363 | molecular_function | heterocyclic compound binding | 1.091 | 3.31E-10 |
| GO:0043231 | cellular_component | intracellular membrane-bounded organelle | 1.171 | 3.31E-10 |
| GO:0044444 | cellular_component | cytoplasmic part | 1.794 | 3.31E-10 |
| GO:0043227 | cellular_component | membrane-bounded organelle | 1.143 | 4.76E-10 |
| GO:0043229 | cellular_component | intracellular organelle | 1.033 | 4.97E-10 |
| GO:0005622 | cellular_component | intracellular | 0.865 | 6.91E-10 |
| GO:0005737 | cellular_component | cytoplasm | 1.753 | 6.91E-10 |
| GO:0043226 | cellular_component | organelle | 1.018 | 7.76E-10 |
| GO:0044763 | biological_process | single-organism cellular process | 1.016 | 1.42E-07 |
| GO:0005623 | cellular_component | cell | 0.578 | 9.55E-06 |
| GO:0044464 | cellular_component | cell part | 0.578 | 9.55E-06 |
| GO:0044699 | biological_process | single-organism process | 0.708 | 3.38E-04 |
| GO:0009987 | biological_process | cellular process | 0.541 | 4.88E-04 |
| GO:0005575 | cellular_component | cellular_component | 0.318 | 4.11E-02 |
| GO:0043189 | cellular_component | H4/H2A histone acetyltransferase complex | 4.248 | 6.01E-02 |
| | | | | |
| **M1** | | | | |
| GOID | Ontology | Term | Log odds-ratio | FDR |
| | | | | |
| **M2** | | | | |
| GOID | Ontology | Term | Log odds-ratio | FDR |
| GO:0005622 | cellular_component | intracellular | 0.58 | 2.14E-02 |
| GO:0044424 | cellular_component | intracellular part | 0.582 | 2.14E-02 |

| | | | | |
|---|---|---|---|---|
| GO:0032991 | cellular_component | macromolecular complex | 1.026 | 2.14E-02 |
| GO:0044422 | cellular_component | organelle part | 1.103 | 2.14E-02 |
| GO:0044446 | cellular_component | intracellular organelle part | 1.132 | 2.14E-02 |
| GO:0043933 | biological_process | macromolecular complex subunit organization | 1.838 | 2.23E-02 |
| GO:0006325 | biological_process | chromatin organization | 2 | 9.43E-02 |
| | | | | |
| **M4** | | | | |
| GOID | Ontology | Term | Log odds-ratio | FDR |
| GO:0000785 | cellular_component | chromatin | 4.178 | 2.25E-25 |
| GO:0034728 | biological_process | nucleosome organization | 4.178 | 2.25E-25 |
| GO:0071824 | biological_process | protein-DNA complex subunit organization | 4.178 | 2.25E-25 |
| GO:0006325 | biological_process | chromatin organization | 3.592 | 4.63E-21 |
| GO:0044427 | cellular_component | chromosomal part | 3.434 | 4.57E-19 |
| GO:0043933 | biological_process | macromolecular complex subunit organization | 3.12 | 4.57E-19 |
| GO:0005694 | cellular_component | chromosome | 3.313 | 8.04E-19 |
| GO:0051276 | biological_process | chromosome organization | 3.123 | 6.95E-18 |
| GO:0006996 | biological_process | organelle organization | 1.997 | 2.44E-10 |
| GO:0043228 | cellular_component | non-membrane-bounded organelle | 1.876 | 5.12E-10 |
| GO:0043232 | cellular_component | intracellular non-membrane-bounded organelle | 1.876 | 5.12E-10 |
| GO:0044422 | cellular_component | organelle part | 1.578 | 5.48E-09 |
| GO:0044446 | cellular_component | intracellular organelle part | 1.582 | 1.50E-08 |
| GO:0071840 | biological_process | cellular component organization or biogenesis | 1.585 | 1.50E-08 |
| GO:0003676 | molecular_function | nucleic acid binding | 1.416 | 3.47E-08 |
| GO:0016043 | biological_process | cellular component organization | 1.594 | 3.52E-08 |
| GO:0044444 | cellular_component | cytoplasmic part | 1.57 | 5.86E-06 |
| GO:0005737 | cellular_component | cytoplasm | 1.529 | 1.09E-05 |
| GO:0043227 | cellular_component | membrane-bounded organelle | 0.945 | 3.52E-05 |
| GO:0043231 | cellular_component | intracellular membrane-bounded organelle | 0.942 | 6.56E-05 |
| GO:0043189 | cellular_component | H4/H2A histone acetyltransferase complex | 5.083 | 6.56E-05 |
| GO:0044424 | cellular_component | intracellular part | 0.689 | 1.49E-04 |
| GO:0005622 | cellular_component | intracellular | 0.668 | 2.66E-04 |
| GO:0043226 | cellular_component | organelle | 0.785 | 3.27E-04 |
| GO:0043229 | cellular_component | intracellular organelle | 0.753 | 1.16E-03 |
| GO:0000123 | cellular_component | histone acetyltransferase complex | 4.183 | 1.53E-03 |
| GO:0044699 | biological_process | single-organism process | 0.698 | 1.55E-03 |
| GO:0044763 | biological_process | single-organism cellular process | 0.797 | 2.21E-03 |
| GO:0005623 | cellular_component | cell | 0.49 | 3.07E-03 |
| GO:0044464 | cellular_component | cell part | 0.49 | 3.07E-03 |
| GO:1901363 | molecular_function | heterocyclic compound binding | 0.741 | 3.97E-03 |

| | | | | |
|---|---|---|---|---|
| GO:0097159 | molecular_function | organic cyclic compound binding | 0.736 | 4.29E-03 |
| GO:0009987 | biological_process | cellular process | 0.503 | 6.25E-03 |
| GO:0005654 | cellular_component | nucleoplasm | 2.624 | 7.65E-03 |
| GO:0044451 | cellular_component | nucleoplasm part | 2.624 | 7.65E-03 |
| GO:0031981 | cellular_component | nuclear lumen | 2.019 | 2.95E-02 |
| GO:0043233 | cellular_component | organelle lumen | 1.924 | 4.72E-02 |
| GO:0070013 | cellular_component | intracellular organelle lumen | 1.924 | 4.72E-02 |
| GO:0031974 | cellular_component | membrane-enclosed lumen | 1.892 | 5.48E-02 |
| GO:0005575 | cellular_component | cellular_component | 0.318 | 7.02E-02 |
| | | | | |
| **S1** | | | | |
| GOID | Ontology | Term | Log odds-ratio | FDR |
| GO:0000785 | cellular_component | chromatin | 4.921 | 2.37E-63 |
| GO:0034728 | biological_process | nucleosome organization | 4.921 | 2.37E-63 |
| GO:0071824 | biological_process | protein-DNA complex subunit organization | 4.921 | 2.37E-63 |
| GO:0006325 | biological_process | chromatin organization | 4.287 | 4.90E-52 |
| GO:0044427 | cellular_component | chromosomal part | 4.177 | 3.04E-50 |
| GO:0051276 | biological_process | chromosome organization | 3.875 | 4.28E-49 |
| GO:0005694 | cellular_component | chromosome | 4.035 | 5.55E-49 |
| GO:0043933 | biological_process | macromolecular complex subunit organization | 3.677 | 4.21E-42 |
| GO:0006996 | biological_process | organelle organization | 2.559 | 1.16E-27 |
| GO:0043228 | cellular_component | non-membrane-bounded organelle | 2.283 | 6.42E-22 |
| GO:0043232 | cellular_component | intracellular non-membrane-bounded organelle | 2.283 | 6.42E-22 |
| GO:0016043 | biological_process | cellular component organization | 2.052 | 9.35E-21 |
| GO:0003676 | molecular_function | nucleic acid binding | 1.822 | 2.55E-20 |
| GO:0071840 | biological_process | cellular component organization or biogenesis | 1.973 | 1.34E-19 |
| GO:0044446 | cellular_component | intracellular organelle part | 1.946 | 8.29E-19 |
| GO:0044422 | cellular_component | organelle part | 1.875 | 8.93E-18 |
| GO:1901363 | molecular_function | heterocyclic compound binding | 1.138 | 5.96E-12 |
| GO:0097159 | molecular_function | organic cyclic compound binding | 1.132 | 6.92E-12 |
| GO:0043231 | cellular_component | intracellular membrane-bounded organelle | 1.143 | 6.99E-10 |
| GO:0043227 | cellular_component | membrane-bounded organelle | 1.094 | 3.42E-09 |
| GO:0044763 | biological_process | single-organism cellular process | 1.03 | 3.90E-08 |
| GO:0043229 | cellular_component | intracellular organelle | 0.929 | 9.37E-08 |
| GO:0005622 | cellular_component | intracellular | 0.772 | 1.47E-07 |
| GO:0043226 | cellular_component | organelle | 0.914 | 1.47E-07 |
| GO:0044424 | cellular_component | intracellular part | 0.76 | 4.57E-07 |
| GO:0009987 | biological_process | cellular process | 0.622 | 3.83E-06 |
| GO:0043189 | cellular_component | H4/H2A histone acetyltransferase complex | 4.957 | 3.74E-05 |

| | | | | |
|---|---|---|---|---|
| GO:0044699 | biological_process | single-organism process | 0.741 | 4.95E-05 |
| GO:0005737 | cellular_component | cytoplasm | 1.309 | 2.72E-04 |
| GO:0044444 | cellular_component | cytoplasmic part | 1.302 | 4.40E-04 |
| GO:0000123 | cellular_component | histone acetyltransferase complex | 4.057 | 9.53E-04 |
| GO:0005623 | cellular_component | cell | 0.46 | 2.32E-03 |
| GO:0044464 | cellular_component | cell part | 0.46 | 2.32E-03 |
| GO:0006397 | biological_process | mRNA processing | 2.413 | 1.07E-02 |
| GO:0005488 | molecular_function | binding | 0.296 | 2.33E-02 |
| GO:0016071 | biological_process | mRNA metabolic process | 2.184 | 2.98E-02 |
| GO:0005575 | cellular_component | cellular_component | 0.29 | 7.16E-02 |
| | | | | |
| **S2** | | | | |
| GOID | Ontology | Term | Log odds-ratio | FDR |
| | | | | |
| **S3** | | | | |
| GOID | Ontology | Term | Log odds-ratio | FDR |
| GO:0035050 | biological_process | embryonic heart tube development | 5.872 | 6.94E-02 |

## Selective sweeps in genes with experimentally verified functions

In the second column, numbers given in parentheses represent the number of genes from the relevant group showing sweep signals (Pool-hmm score >4) in each population.

| Gene(s) | Populations with Pool-hmm score>4 | Adaptive phenotype | References |
|---|---|---|---|
| Histones | A1(76), A4(77), M1(63), M2(77), M4(76), S1(79), S2(77), S3(34) | Regulation of genome-wide or regional gene expression | *See the section on histones later in this article.* |
| Chromatin remodeling factors | | Regulation of genome-wide or regional gene expression | [1–6] |
| Histone acetyltransferase PCAF | A1, A4, M1, S1, S2 | | |
| Histone deacetylases* | A1(2), M1(3), M2(4), M4(1), S1(2), S2(1) | | |
| Histone methyltransferases* | A1(1), A4(1), M1(2), M2(2), M4(1), S1(2), S2(2) | | |
| Histone demethylase* | S3(1) | | |
| Chromatin assembly factor 1, p-180 subunit | A1, A4, M1, M2, S1, S2, S3 | | |
| ATP-dependent chromatin assembly factor large subunit | A1, M1, M2, S1, S2 | | |
| Heterochromatin protein 1 | A1, S2, S3 | | |
| Chromatin regulatory protein sir2 | M2(1), M4(1) | | |
| P450 cytochrome family | A1(1), M1(65), M2(11), M4(7), S1(13), S2(12), S3(18) | Insecticide resistance, Insect hormone biosynthesis (KEGG pathway #cqu00981) | [7–12] |
| Ribosomal proteins | A1(31), A4(32), M1(57), M2(19), M4(17), S1(70), S2(52), S3(63) | Insecticide resistance, Induction of diapause | [13–18] |
| Chaperonins and heat shock proteins | A1(14), A4(14), M1(21), M2(4), M4(1), S1(28), S2(26), S3(36) | Adaptation to high and low temperatures e.g. cold-resistance during diapause | [19–23] |
| Vitellogenins and vitellogenin convertase | A1(2), M1(4), M2(5), M4(2), S1(1), S2(1), S3(1) | Regulation of female reproduction | [24,25] |
| Cadherins | A1(11), A4(11), M1(13), M2(4), M4(1), S1(21), S2(21), S3(19) | Insecticide resistance, Fertility | [26,27] |
| Superoxide dismutases | A1(4), A4(2), M1(2), M2(1), S1(5), S2(5), S3(7) | Protection of ovaries and survival during diapause | [28] |
| Salivary proteins | M1(17), M2(4), M4(1), S1(4), S3(2) | Host immune response modulation | [29] |

* Only genes named as histone deactylase, histone methyltransferase or histone demethylase were counted. General deactylases, methyltransferases or methylases which may act on histones also, were not included.

**References:**


1. Pecinka, A. & Mittelsten Scheid, O. 2012 Stress-induced chromatin changes: a critical view on their heritability. *Plant Cell Physiol.* **53**, 801–8. (doi:10.1093/pcp/pcs044)

2. Hirsch, S., Baumberger, R. & Grossniklaus, U. 2012 Epigenetic variation, inheritance, and selection in plant populations. *Cold Spring Harb. Symp. Quant. Biol.* **77**, 97–104. (doi:10.1101/sqb.2013.77.014605)

3. Richards, E. J. 2008 Population epigenetics. *Curr. Opin. Genet. Dev.* **18**, 221–6. (doi:10.1016/j.gde.2008.01.014)

4. Levine, M. T., Eckert, M. L. & Begun, D. J. 2011 Whole-genome expression plasticity across tropical and temperate Drosophila melanogaster populations from Eastern Australia. *Mol. Biol. Evol.* **28**, 249–56. (doi:10.1093/molbev/msq197)

5. Levine, M. T. & Begun, D. J. 2008 Evidence of spatially varying selection acting on four chromatin-remodeling loci in Drosophila melanogaster. *Genetics* **179**, 475–85. (doi:10.1534/genetics.107.085423)

6. Kolaczkowski, B., Kern, A. D., Holloway, A. K. & Begun, D. J. 2011 Genomic differentiation between temperate and tropical Australian populations of Drosophila melanogaster. *Genetics* **187**, 245–60. (doi:10.1534/genetics.110.123059)

7. Sun, Y. et al. 2012 Functional characterization of an arrestin gene on insecticide resistance of Culex pipiens pallens. *Parasit. Vectors* **5**, 134. (doi:10.1186/1756-3305-5-134)

8. Shen, B., Dong, H.-Q., Tian, H.-S., Ma, L., Li, X.-L., Wu, G.-L. & Zhu, C.-L. 2003 Cytochrome P450 genes expressed in the deltamethrin-susceptible and -resistant strains of Culex pipiens pallens. *Pestic. Biochem. Physiol.* **75**, 19–26. (doi:10.1016/S0048-3575(03)00014-2)

9. Yan, L., Yang, P., Jiang, F., Cui, N., Ma, E., Qiao, C. & Cui, F. 2012 Transcriptomic and phylogenetic analysis of Culex pipiens quinquefasciatus for three detoxification gene families. *BMC Genomics* **13**, 609. (doi:10.1186/1471-2164-13-609)

10. Reddy, B. N., Rao, B. P., Prasad, G. & Raghavendra, K. 2012 Identification and classification of detoxification enzymes from Culex quinquefasciatus (Diptera: Culicidae). *Bioinformation* **8**, 430–6. (doi:10.6026/97320630008430)

11. Itokawa, K., Komagata, O., Kasai, S., Kawada, H., Mwatele, C., Dida, G. O., Njenga, S. M., Mwandawiro, C. & Tomita, T. 2013 Global spread and genetic variants of the two CYP9M10 haplotype forms associated with insecticide resistance in Culex quinquefasciatus Say. *Heredity (Edinb).* **111**, 216–26. (doi:10.1038/hdy.2013.40)

12. Kanehisa, M., Goto, S., Sato, Y., Furumichi, M. & Tanabe, M. 2012 KEGG for integration and interpretation of large-scale molecular data sets. *Nucleic Acids Res.* **40**, D109–14. (doi:10.1093/nar/gkr988)

13. Kim, M. & Denlinger, D. L. 2010 A potential role for ribosomal protein S2 in the gene network regulating reproductive diapause in the mosquito Culex pipiens. *J. Comp. Physiol. B.* **180**, 171–8. (doi:10.1007/s00360-009-0406-9)

14. Kim, M., Sim, C. & Denlinger, D. L. 2010 RNA interference directed against ribosomal protein S3a suggests a link between this gene and arrested ovarian development during adult diapause in Culex pipiens. *Insect Mol. Biol.* **19**, 27–33. (doi:10.1111/j.1365-2583.2009.00926.x.RNA)

15. He, J. et al. 2009 Cloning and characterization of 60S ribosomal protein L22 (RPL22) from Culex pipiens pallens. *Comp. Biochem. Physiol. B. Biochem. Mol. Biol.* **153**, 216–22. (doi:10.1016/j.cbpb.2009.03.003)

16. Hu, X., Wang, W., Zhang, D., Jiao, J., Tan, W., Sun, Y., Ma, L. & Zhu, C. 2007 Cloning and characterization of 40S ribosomal protein S4 gene from Culex pipiens pallens. *Comp. Biochem. Physiol. B. Biochem. Mol. Biol.* **146**, 265–70. (doi:10.1016/j.cbpb.2006.11.011)

17. Tan, W. et al. 2006 Cloning and overexpression of ribosomal protein L39 gene from deltamethrin-resistant Culex pipiens pallens. *Exp. Parasitol.* **115**, 369–78.

18. Sun, H. et al. 2011 Cloning and characterization of ribosomal protein S29, a deltamethrin resistance associated gene from Culex pipiens pallens. *Parasitol. Res.* **109**, 1689–97. (doi:10.1007/s00436-011-2443-z)

19. Ferrer, M., Chernikova, T. N., Yakimov, M. M., Golyshin, P. N. & Timmis, K. N. 2003 Chaperonins govern growth of Escherichia coli at low temperatures. *Nat. Biotechnol.* **21**, 1266–1267.

20. Fujiwara, S., Aki, R., Yoshida, M., Higashibata, H., Imanaka, T. & Fukuda, W. 2008 Expression profiles and physiological roles of two types of molecular chaperonins from the hyperthermophilic archaeon Thermococcus kodakarensis. *Appl. Environ. Microbiol.* **74**, 7306–12. (doi:10.1128/AEM.01245-08)

21. Somer, L., Shmulman, O., Dror, T., Hashmueli, S. & Kashi, Y. 2002 The eukaryote chaperonin CCT is a cold shock protein in Saccharomyces cerevisiae. *Cell Stress Chaperones* **7**, 47–54.

22. Kayukawa, T., Chen, B., Miyazaki, S., Itoyama, K., Shinoda, T. & Ishikawa, Y. 2005 Expression of mRNA for the t-complex polypeptide-1, a subunit of chaperonin CCT, is upregulated in association with increased cold hardiness in Delia antiqua. *Cell Stress Chaperones* **10**, 204–10.

23. Kayukawa, T. & Ishikawa, Y. 2009 Chaperonin contributes to cold hardiness of the onion maggot Delia antiqua through repression of depolymerization of actin at low temperatures. *PLoS One* **4**, e8277. (doi:10.1371/journal.pone.0008277)

24. Provost-Javier, K. N., Chen, S. & Rasgon, J. L. 2010 Vitellogenin gene expression in autogenous Culex tarsalis. *Insect Mol. Biol.* **19**, 423–9. (doi:10.1111/j.1365-2583.2010.00999.x)

25. Raikhel, A. S. et al. 2002 Molecular biology of mosquito vitellogenesis: from basic studies to genetic engineering of antipathogen immunity. *Insect Biochem. Mol. Biol.* **32**, 1275–86.

26. Morin, S. et al. 2003 Three cadherin alleles associated with resistance to Bacillus thuringiensis in pink bollworm. *Proc. Natl. Acad. Sci. U. S. A.* **100**, 5004–9. (doi:10.1073/pnas.0831036100)

27. Zhao, J., Jin, L., Yang, Y. & Wu, Y. 2010 Diverse cadherin mutations conferring resistance to Bacillus thuringiensis toxin Cry1Ac in Helicoverpa armigera. *Insect Biochem. Mol. Biol.* **40**, 113–8. (doi:10.1016/j.ibmb.2010.01.001)

28. Sim, C. & Denlinger, D. L. 2011 Catalase and superoxide dismutase-2 enhance survival and protect ovaries during overwintering diapause in the mosquito Culex pipiens. *J. Insect Physiol.* **57**, 628–34. (doi:10.1016/j.jinsphys.2011.01.012)

29. Calvo, E., Mans, B. J., Andersen, J. F. & Ribeiro, J. M. C. 2006 Function and evolution of a mosquito salivary protein family. *J. Biol. Chem.* **281**, 1935–42. (doi:10.1074/jbc.M510359200)

## Supplementary file 5: Polymorphism pattern in histones

The overall polymorphism is lower in the histone block compared to the background genome (only non-sweep genes included). Generally, histone H1 has a polymorphism level higher than the more conserved nucleosomal histones but lower than the background. The ratio of Nsyn/Syn, however, is similar for H1 and other histones, and is remarkably higher than that of background.

| | A1 | A4 | M1 | M2 | M4 | S1 | S2 | S3 |
|---|---|---|---|---|---|---|---|---|
| **Per gene Syn, Hist block** | 1.688 | 3.712 | 1.588 | 1.35 | 2.138 | 3.75 | 0.65 | 0.462 |
| **Per gene Nsyn, Hist block** | 2.425 | 4.4625 | 2.0125 | 1.4625 | 1.9125 | 5.575 | 0.8375 | 0.4 |
| **Nsyn/Syn, Hist block** | 1.437 | 1.202 | 1.268 | 1.083 | 0.895 | 1.487 | 1.288 | 0.865 |
| **Per gene total, Hist block** | 4.1125 | 8.175 | 3.6 | 2.8125 | 4.05 | 9.325 | 1.4875 | 0.8625 |
| | | | | | | | | |
| **Per gene Syn, H1 in block** | 6.333 | 9.389 | 5.667 | 4.722 | 4.556 | 12.111 | 2.5 | 2 |
| **Per gene Nsyn, H1 in block** | 8.389 | 13 | 6.833 | 6.056 | 5.222 | 16.5 | 3.389 | 1.611 |
| **Nsyn/Syn, H1 in block** | 1.324 | 1.3846 | 1.206 | 1.282 | 1.146 | 1.362 | 1.355 | 0.806 |
| **Per gene total, H1 in block** | 14.722 | 22.389 | 12.5 | 10.778 | 9.778 | 28.611 | 5.889 | 3.611 |
| | | | | | | | | |
| **Per gene Syn in background** | 22.18 | 21.84 | 20.4 | 11.41 | 4.56 | 21.56 | 31.59 | 29.99 |
| **Per gene Nsyn in background** | 10.34 | 9.05 | 13.07 | 8.9 | 3.2 | 8.56 | 15.24 | 16.6 |
| **Nsyn/Syn in background** | 0.466 | 0.414 | 0.641 | 0.780 | 0.702 | 0.397 | 0.482 | 0.554 |
| **Per gene total in background** | 32.52 | 30.88 | 33.47 | 20.31 | 7.76 | 30.12 | 46.83 | 46.58 |

There are four histone H1 genes outside our focal 137kb block (CPIJ010358, CPIJ010363, CPIJ14770 and CPIJ018062). The table below presents the polymorphism data for H1 genes within the 137kb block and these four outsider genes. (CPIJ010363 has a sweep score>4 in M1, and CPIJ018062 has sweep scores>4 in M1 and S2. These 3 cases were excluded from calculations.) Evidently, the non-sweep H1 loci outside the block have more synonymous than nonsynonymous polymorphisms, in contrast to their counterparts within the swept block.

| | **Syn** | **Nsyn** |
|---|---|---|
| **H1 in the block** | 851 | 1098 |
| **H1 outside the block** | 352 | 322 |
| Chi-square=14.79 | P<0.0001 | |

# Supplementary file 6: Biochemical vs structural aspects of histone H1 polymorphisms

**Description of variables:**

Nsyn_YN: =1 if the residue is nonsynonymously polymorphic in at least one of the 8 populations; =0 otherwise.

Domains: C=C-terminal, G=globular, N=N-terminal

Secondary structures: E=extended (beta strand), H=helix, - = not E or H

B25: =B if less than 25% solvent accessibility; = - otherwise.

ST_YN: =1 if the residue allows addition or removal of Ser/Thr in at least one of the 8 populations; =0 otherwise.

Pro_abs: =the total number of populations with a +/- proline mutation at the given position.

| Table of Domain by Nsyn_YN | | | |
|---|---|---|---|
| Domain(Domain) | Nsyn_YN | | |
| Frequency<br>Percent<br>Row Pct<br>Col Pct | 0 | 1 | Total |
| C | 1373<br>40.75<br>78.41<br>49.16 | 378<br>11.22<br>21.59<br>65.63 | 1751<br>51.97 |
| G | 995<br>29.53<br>94.76<br>35.62 | 55<br>1.63<br>5.24<br>9.55 | 1050<br>31.17 |
| N | 425<br>12.62<br>74.82<br>15.22 | 143<br>4.24<br>25.18<br>24.83 | 568<br>16.86 |
| Total | 2793<br>82.90 | 576<br>17.10 | 3369<br>100.00 |

***Statistics for Table of Domain by Nsyn_YN***

| Statistic | DF | Value | Prob |
|---|---|---|---|
| Chi-Square | 2 | 155.2495 | <.0001 |
| Likelihood Ratio Chi-Square | 2 | 182.8464 | <.0001 |
| Mantel-Haenszel Chi-Square | 1 | 3.9720 | 0.0463 |
| Phi Coefficient | | 0.2147 | |
| Contingency Coefficient | | 0.2099 | |
| Cramer's V | | 0.2147 | |

***Sample Size = 3369***

| Table of Secondary by Nsyn_YN | | | |
|---|---|---|---|
| Secondary(Secondary) | Nsyn_YN | | |
| Frequency<br>Percent<br>Row Pct<br>Col Pct | 0 | 1 | Total |
| - | 2071<br>61.47<br>78.87<br>74.15 | 555<br>16.47<br>21.13<br>96.35 | 2626<br>77.95 |
| E | 119<br>3.53<br>96.75<br>4.26 | 4<br>0.12<br>3.25<br>0.69 | 123<br>3.65 |
| H | 603<br>17.90<br>97.26<br>21.59 | 17<br>0.50<br>2.74<br>2.95 | 620<br>18.40 |
| Total | 2793<br>82.90 | 576<br>17.10 | 3369<br>100.00 |

***Statistics for Table of Secondary by Nsyn_YN***

| Statistic | DF | Value | Prob |
|---|---|---|---|
| Chi-Square | 2 | 136.9787 | <.0001 |
| Likelihood Ratio Chi-Square | 2 | 182.3703 | <.0001 |
| Mantel-Haenszel Chi-Square | 1 | 130.7704 | <.0001 |
| Phi Coefficient | | 0.2016 | |
| Contingency Coefficient | | 0.1977 | |
| Cramer's V | | 0.2016 | |

***Sample Size = 3369***

| Table of B25 by Nsyn_YN | | | |
|---|---|---|---|
| B25(B25) | Nsyn_YN | | |
| Frequency<br>Percent<br>Row Pct<br>Col Pct | 0 | 1 | Total |
| - | 2108<br>62.57<br>80.43<br>75.47 | 513<br>15.23<br>19.57<br>89.06 | 2621<br>77.80 |
| B | 685<br>20.33<br>91.58<br>24.53 | 63<br>1.87<br>8.42<br>10.94 | 748<br>22.20 |
| Total | 2793<br>82.90 | 576<br>17.10 | 3369<br>100.00 |

***Statistics for Table of B25 by Nsyn_YN***

| Statistic | DF | Value | Prob |
|---|---|---|---|
| Chi-Square | 1 | 51.0438 | <.0001 |
| Likelihood Ratio Chi-Square | 1 | 58.0570 | <.0001 |
| Continuity Adj. Chi-Square | 1 | 50.2602 | <.0001 |
| Mantel-Haenszel Chi-Square | 1 | 51.0287 | <.0001 |
| Phi Coefficient | | -0.1231 | |
| Contingency Coefficient | | 0.1222 | |
| Cramer's V | | -0.1231 | |

| Fisher's Exact Test | |
|---|---|
| Cell (1,1) Frequency (F) | 2108 |
| Left-sided Pr <= F | 2.223E-14 |
| Right-sided Pr >= F | 1.0000 |
| | |
| Table Probability (P) | 1.395E-14 |
| Two-sided Pr <= P | 4.063E-14 |

***Sample Size = 3369***

| Table of B25 by Charge_YN | | | |
|---|---|---|---|
| B25(B25) | Charge_YN | | |
| Frequency<br>Percent<br>Row Pct<br>Col Pct | 0 | 1 | Total |
| - | 2494<br>74.03<br>95.15<br>77.05 | 127<br>3.77<br>4.85<br>96.21 | 2621<br>77.80 |
| B | 743<br>22.05<br>99.33<br>22.95 | 5<br>0.15<br>0.67<br>3.79 | 748<br>22.20 |
| Total | 3237<br>96.08 | 132<br>3.92 | 3369<br>100.00 |

***Statistics for Table of B25 by Charge_YN***

| Statistic | DF | Value | Prob |
|---|---|---|---|
| Chi-Square | 1 | 26.9704 | <.0001 |
| Likelihood Ratio Chi-Square | 1 | 37.3255 | <.0001 |
| Continuity Adj. Chi-Square | 1 | 25.8723 | <.0001 |
| Mantel-Haenszel Chi-Square | 1 | 26.9624 | <.0001 |
| Phi Coefficient | | -0.0895 | |
| Contingency Coefficient | | 0.0891 | |
| Cramer's V | | -0.0895 | |

| Fisher's Exact Test | |
|---|---|
| Cell (1,1) Frequency (F) | 2494 |
| Left-sided Pr <= F | 1.622E-09 |
| Right-sided Pr >= F | 1.0000 |
| | |
| Table Probability (P) | 1.416E-09 |
| Two-sided Pr <= P | 2.837E-09 |

***Sample Size = 3369***

| Table of B25 by Pro_YN | | | |
|---|---|---|---|
| B25(B25) | Pro_YN | | |
| Frequency<br>Percent<br>Row Pct<br>Col Pct | 0 | 1 | Total |
| - | 2390<br>70.94<br>91.19<br>76.87 | 231<br>6.86<br>8.81<br>88.85 | 2621<br>77.80 |
| B | 719<br>21.34<br>96.12<br>23.13 | 29<br>0.86<br>3.88<br>11.15 | 748<br>22.20 |
| Total | 3109<br>92.28 | 260<br>7.72 | 3369<br>100.00 |

***Statistics for Table of B25 by Pro_YN***

| Statistic | DF | Value | Prob |
|---|---|---|---|
| Chi-Square | 1 | 19.9113 | <.0001 |
| Likelihood Ratio Chi-Square | 1 | 22.9453 | <.0001 |
| Continuity Adj. Chi-Square | 1 | 19.2242 | <.0001 |
| Mantel-Haenszel Chi-Square | 1 | 19.9054 | <.0001 |
| Phi Coefficient | | -0.0769 | |
| Contingency Coefficient | | 0.0767 | |
| Cramer's V | | -0.0769 | |

| Fisher's Exact Test | |
|---|---|
| Cell (1,1) Frequency (F) | 2390 |
| Left-sided Pr <= F | 1.405E-06 |
| Right-sided Pr >= F | 1.0000 |
| | |
| Table Probability (P) | 8.371E-07 |
| Two-sided Pr <= P | 2.620E-06 |

***Sample Size = 3369***

The calculations in the table below address this question:

Given a residue is known to allow nonsynonymous polymorphism, will it be less permissive to charge alteration if it is buried in the depth of the protein? Comparing the closeness of 46.08% and 45.02%, with the big difference between 24.78% and 7.96%, the answer is YES.

| | **Buried** | **Exposed** | **Source (this file)** |
|---|---|---|---|
| **N=% allow Nsyn change** | 8.42 | 19.57 | Page 4 |
| **C=% allow charge alteration** | 0.67 | 4.85 | Page 5 |
| **P=% allow +/- proline** | 3.88 | 8.81 | Page 6 |
| **% C/N** | 7.96 | 24.78 | |
| **% P/N** | 46.08 | 45.02 | |

| Table of B25 by ST_YN | | | |
|---|---|---|---|
| B25(B25) | ST_YN | | |
| Frequency<br>Percent<br>Row Pct<br>Col Pct | 0 | 1 | Total |
| - | 2490<br>73.91<br>95.00<br>77.26 | 131<br>3.89<br>5.00<br>89.73 | 2621<br>77.80 |
| B | 733<br>21.76<br>97.99<br>22.74 | 15<br>0.45<br>2.01<br>10.27 | 748<br>22.20 |
| Total | 3223<br>95.67 | 146<br>4.33 | 3369<br>100.00 |

***Statistics for Table of B25 by ST_YN***

| Statistic | DF | Value | Prob |
|---|---|---|---|
| Chi-Square | 1 | 12.5718 | 0.0004 |
| Likelihood Ratio Chi-Square | 1 | 14.7985 | 0.0001 |
| Continuity Adj. Chi-Square | 1 | 11.8602 | 0.0006 |
| Mantel-Haenszel Chi-Square | 1 | 12.5680 | 0.0004 |
| Phi Coefficient | | -0.0611 | |
| Contingency Coefficient | | 0.0610 | |
| Cramer's V | | -0.0611 | |

| Fisher's Exact Test | |
|---|---|
| Cell (1,1) Frequency (F) | 2490 |
| Left-sided Pr <= F | 1.064E-04 |
| Right-sided Pr >= F | 1.0000 |
| | |
| Table Probability (P) | 6.705E-05 |
| Two-sided Pr <= P | 2.146E-04 |

***Sample Size = 3369***

| Table of Domain by Pro_YN | | | |
|---|---|---|---|
| Domain(Domain) | Pro_YN | | |
| Frequency<br>Percent<br>Row Pct<br>Col Pct | 0 | 1 | Total |
| C | 1576<br>46.78<br>90.01<br>50.69 | 175<br>5.19<br>9.99<br>67.31 | 1751<br>51.97 |
| G | 1022<br>30.34<br>97.33<br>32.87 | 28<br>0.83<br>2.67<br>10.77 | 1050<br>31.17 |
| N | 511<br>15.17<br>89.96<br>16.44 | 57<br>1.69<br>10.04<br>21.92 | 568<br>16.86 |
| Total | 3109<br>92.28 | 260<br>7.72 | 3369<br>100.00 |

***Statistics for Table of Domain by Pro_YN***

| Statistic | DF | Value | Prob |
|---|---|---|---|
| Chi-Square | 2 | 54.6410 | <.0001 |
| Likelihood Ratio Chi-Square | 2 | 65.0931 | <.0001 |
| Mantel-Haenszel Chi-Square | 1 | 5.2580 | 0.0218 |
| Phi Coefficient | | 0.1274 | |
| Contingency Coefficient | | 0.1263 | |
| Cramer's V | | 0.1274 | |

***Sample Size = 3369***

| Table of Domain by Pro_abs | | | | | | | | |
|---|---|---|---|---|---|---|---|---|
| Domain(Domain) | Pro_abs | | | | | | | |
| Frequency<br>Percent<br>Row Pct<br>Col Pct | 0 | 1 | 2 | 3 | 4 | 5 | 6 | Total |
| C | 1576<br>46.78<br>90.01<br>50.69 | 116<br>3.44<br>6.62<br>68.64 | 27<br>0.80<br>1.54<br>60.00 | 20<br>0.59<br>1.14<br>68.97 | 10<br>0.30<br>0.57<br>71.43 | 2<br>0.06<br>0.11<br>100.00 | 0<br>0.00<br>0.00<br>0.00 | 1751<br>51.97 |
| G | 1022<br>30.34<br>97.33<br>32.87 | 6<br>0.18<br>0.57<br>3.55 | 11<br>0.33<br>1.05<br>24.44 | 6<br>0.18<br>0.57<br>20.69 | 4<br>0.12<br>0.38<br>28.57 | 0<br>0.00<br>0.00<br>0.00 | 1<br>0.03<br>0.10<br>100.00 | 1050<br>31.17 |
| N | 511<br>15.17<br>89.96<br>16.44 | 47<br>1.40<br>8.27<br>27.81 | 7<br>0.21<br>1.23<br>15.56 | 3<br>0.09<br>0.53<br>10.34 | 0<br>0.00<br>0.00<br>0.00 | 0<br>0.00<br>0.00<br>0.00 | 0<br>0.00<br>0.00<br>0.00 | 568<br>16.86 |
| Total | 3109<br>92.28 | 169<br>5.02 | 45<br>1.34 | 29<br>0.86 | 14<br>0.42 | 2<br>0.06 | 1<br>0.03 | 3369<br>100.00 |

***Statistics for Table of Domain by Pro_abs***

| Statistic | DF | Value | Prob |
|---|---|---|---|
| Chi-Square | 12 | 78.7084 | <.0001 |
| Likelihood Ratio Chi-Square | 12 | 105.0427 | <.0001 |
| Mantel-Haenszel Chi-Square | 1 | 8.1461 | 0.0043 |
| Phi Coefficient | | 0.1528 | |
| Contingency Coefficient | | 0.1511 | |
| Cramer's V | | 0.1081 | |
| WARNING: 43% of the cells have expected counts less than 5. Chi-Square may not be a valid test. | | | |

***Sample Size = 3369***

Among the residues allowing Pro mutations, the proportion showing Pro mutations in more than one population for each of the 3 domains:

C: 59/175=33.71% G: 22/28=78.57% N: 10/57=17.54%

# Supplementary file 7: Independence of polymorphic positions between *C. torrentium* and *C. pipiens*

Description of variables:

%sample_Nsyn: =1 if the residue shows nonsynonymous polymorphism in the corresponding sample; =0 otherwise.

%sample_Pro: =+1 if a mutation converts an alternative reference residue to proline; = -1 if a mutation converts the reference proline residue to a different aminoacid; =0 otherwise.

## P-values associated with Fisher's exact test for independence of positions of nonsynonymous polymorphisms

| Fisher's exact test, N = 3369, Total probability (P) Pr <= P | | | | | | | | |
|---|---|---|---|---|---|---|---|---|
| | **A1_Nsyn** | **A4_Nsyn** | **M1_Nsyn** | **M2_Nsyn** | **M4_Nsyn** | **S1_Nsyn** | **S2_Nsyn** | **S3_Nsyn** |
| **A1_Nsyn** | 4.19E-220<br>1.07E-225 | 4.751E-23<br>5.060E-24 | 8.184E-43<br>2.012E-44 | 0.1601<br>0.3627 | 0.2310<br>1.0000 | 1.538E-18<br>2.164E-19 | 1.775E-27<br>5.391E-29 | 7.907E-11<br>8.269E-11 |
| **A4_Nsyn** | | 0.0000<br>0.0000 | 1.166E-13<br>1.329E-13 | 1.311E-05<br>1.691E-05 | 0.0030<br>0.0044 | 6.188E-38<br>6.369E-39 | 0.0026<br>0.0036 | 4.947E-08<br>5.388E-08 |
| **M1_Nsyn** | | | 1.76E-172<br>6.02E-178 | 0.0032<br>0.0041 | 0.2471<br>0.7247 | 5.695E-11<br>6.737E-11 | 7.868E-21<br>2.772E-22 | 1.665E-15<br>1.592E-15 |
| **M2_Nsyn** | | | | 5.11E-155<br>1.99E-160 | 7.581E-05<br>8.954E-05 | 4.158E-05<br>5.545E-05 | 0.2464<br>0.6493 | 0.5055<br>1.0000 |
| **M4_Nsyn** | | | | | 1.92E-161<br>7.14E-167 | 2.099E-05<br>2.788E-05 | 0.2250<br>0.4033 | 0.4879<br>1.0000 |
| **S1_Nsyn** | | | | | | 0.0000<br>0.0000 | 1.030E-10<br>1.170E-10 | 4.531E-04<br>5.415E-04 |
| **S2_Nsyn** | | | | | | | 6.27E-125<br>3.16E-130 | 2.474E-16<br>4.092E-18 |
| **S3_Nsyn** | | | | | | | | 4.871E-67<br>5.029E-72 |

Note: Since 28 pairwise comparisons are made, the significance threshold should be considered 0.05/28=0.0018 after Bonferroni correction.

Nonsignificant tests indicate independent distribution of nonsynonymous positions in histone H1 residues between the compared populations. The proportion of non-significant tests (highlighted on the upper half of the table):

For intraspecific comparisons: 1/16.

For interspecific comparisons: 9/12.

**P-values associated with Fisher's exact test for independence of positions of proline polymorphisms**

| Fisher's exact test, N = 3369, Total probability (P)<br>Pr <= P | | | | | | | | |
|---|---|---|---|---|---|---|---|---|
| | **A1_Pro** | **A4_Pro** | **M1_Pro** | **M2_Pro** | **M4_Pro** | **S1_Pro** | **S2_Pro** | **S3_Pro** |
| **A1_Pro** | 6.66E-115<br>6.66E-115 | 2.371E-27<br>2.221E-26 | 5.173E-37<br>1.830E-36 | 0.2508<br>0.4027 | 0.0506<br>0.0828 | 2.852E-19<br>6.624E-18 | 2.328E-14<br>3.839E-13 | 8.659E-11<br>3.743E-10 |
| **A4_Pro** | | 4.701E-212<br>4.701E-212 | 2.044E-18<br>1.905E-17 | 0.0014<br>0.0094 | 1.332E-06<br>4.677E-06 | 1.257E-42<br>3.889E-41 | 1.334E-06<br>1.779E-05 | 6.264E-10<br>3.021E-09 |
| **M1_Pro** | | | 3.932E-108<br>3.932E-108 | 1.425E-04<br>7.671E-04 | 0.2595<br>0.3299 | 2.376E-10<br>4.736E-09 | 2.177E-13<br>2.051E-12 | 6.238E-11<br>2.546E-10 |
| **M2_Pro** | | | | 1.310E-89<br>1.310E-89 | 0.0018<br>0.0037 | 1.860E-04<br>0.0021 | 0.0011<br>0.0057 | 0.8637<br>1.0000 |
| **M4_Pro** | | | | | 4.871E-67<br>5.029E-72 | 1.057E-07<br>3.769E-07 | 0.7980<br>1.0000 | 0.8858<br>1.0000 |
| **S1_Pro** | | | | | | 2.928E-223<br>2.928E-223 | 7.882E-08<br>1.444E-06 | 4.601E-04<br>0.0015 |
| **S2_Pro** | | | | | | | 1.302E-70<br>1.302E-70 | 4.391E-11<br>2.017E-10 |
| **S3_Pro** | | | | | | | | 2.636E-40<br>2.636E-40 |

Note: Since 28 pairwise comparisons are made, the significance threshold should be considered 0.05/28=0.0018 after Bonferroni correction.

Nonsignificant tests indicate independent distribution of nonsynonymous positions adding or removing proline in histone H1 residues between the compared populations. The proportion of non-significant tests (highlighted on the upper half of the table):

For intraspecific comparisons: 1/16.

For interspecific comparisons: 9/12.

## Correlation analysis of nonsynonymous polymorphic sites across populations

| Spearman Correlation Coefficients, N = 3369<br>Prob > \|r\| under H0: Rho=0 | | | | | | | | |
|---|---|---|---|---|---|---|---|---|
| | **A1_Nsyn** | **A4_Nsyn** | **M1_Nsyn** | **M2_Nsyn** | **M4_Nsyn** | **S1_Nsyn** | **S2_Nsyn** | **S3_Nsyn** |
| **A1_Nsyn** | 1.00000 | 0.23978<br><.0001 | 0.41580<br><.0001 | 0.01271<br>0.4607 | 0.00057<br>0.9737 | 0.20579<br><.0001 | 0.33583<br><.0001 | 0.18922<br><.0001 |
| **A4_Nsyn** | 0.23978<br><.0001 | 1.00000 | 0.17131<br><.0001 | 0.09054<br><.0001 | 0.05457<br>0.0015 | 0.29738<br><.0001 | 0.05782<br>0.0008 | 0.13505<br><.0001 |
| **M1_Nsyn** | 0.41580<br><.0001 | 0.17131<br><.0001 | 1.00000 | 0.06079<br>0.0004 | -0.01401<br>0.4164 | 0.14524<br><.0001 | 0.29968<br><.0001 | 0.26515<br><.0001 |
| **M2_Nsyn** | 0.01271<br>0.4607 | 0.09054<br><.0001 | 0.06079<br>0.0004 | 1.00000 | 0.09096<br><.0001 | 0.08285<br><.0001 | 0.00912<br>0.5968 | -0.01435<br>0.4052 |
| **M4_Nsyn** | 0.00057<br>0.9737 | 0.05457<br>0.0015 | -0.01401<br>0.4164 | 0.09096<br><.0001 | 1.00000 | 0.08636<br><.0001 | -0.02127<br>0.2171 | -0.01472<br>0.3931 |
| **S1_Nsyn** | 0.20579<br><.0001 | 0.29738<br><.0001 | 0.14524<br><.0001 | 0.08285<br><.0001 | 0.08636<br><.0001 | 1.00000 | 0.14950<br><.0001 | 0.07617<br><.0001 |
| **S2_Nsyn** | 0.33583<br><.0001 | 0.05782<br>0.0008 | 0.29968<br><.0001 | 0.00912<br>0.5968 | -0.02127<br>0.2171 | 0.14950<br><.0001 | 1.00000 | 0.32757<br><.0001 |
| **S3_Nsyn** | 0.18922<br><.0001 | 0.13505<br><.0001 | 0.26515<br><.0001 | -0.01435<br>0.4052 | -0.01472<br>0.3931 | 0.07617<br><.0001 | 0.32757<br><.0001 | 1.00000 |

Note: Since 28 pairwise comparisons are made, the significance threshold should be considered 0.05/28=0.0018 after Bonferroni correction.

Nonsignificant correlations indicate independent distribution of nonsynonymous positions in histone H1 residues between the compared populations. The proportion of non-significant correlations (highlighted on the upper half of the table):

For intraspecific comparisons: 0/16.

For interspecific comparisons: 7/12.

Notice that correlations are always positive when they are significant.

## Correlation analysis of proline polymorphic sites across populations

| Spearman Correlation Coefficients, N = 3369<br>Prob > \|r\| under H0: Rho=0 | | | | | | | | |
|---|---|---|---|---|---|---|---|---|
| | **A1_Pro** | **A4_Pro** | **M1_Pro** | **M2_Pro** | **M4_Pro** | **S1_Pro** | **S2_Pro** | **S3_Pro** |
| **A1_Pro** | 1.00000 | 0.33485<br><.0001 | 0.49999<br><.0001 | 0.01659<br>0.3357 | 0.04370<br>0.0112 | 0.25847<br><.0001 | 0.27686<br><.0001 | 0.26356<br><.0001 |
| **A4_Pro** | 0.33485<br><.0001 | 1.00000 | 0.25862<br><.0001 | 0.07098<br><.0001 | 0.13079<br><.0001 | 0.38687<br><.0001 | 0.12594<br><.0001 | 0.20090<br><.0001 |
| **M1_Pro** | 0.49999<br><.0001 | 0.25862<br><.0001 | 1.00000 | 0.06716<br><.0001 | 0.01766<br>0.3055 | 0.16754<br><.0001 | 0.25617<br><.0001 | 0.27212<br><.0001 |
| **M2_Pro** | 0.01659<br>0.3357 | 0.07098<br><.0001 | 0.06716<br><.0001 | 1.00000 | 0.08811<br><.0001 | 0.08575<br><.0001 | 0.06233<br>0.0003 | -0.00405<br>0.8141 |
| **M4_Pro** | 0.04370<br>0.0112 | 0.13079<br><.0001 | 0.01766<br>0.3055 | 0.08811<br><.0001 | 1.00000 | 0.14585<br><.0001 | -0.00506<br>0.7692 | -0.00516<br>0.7647 |
| **S1_Pro** | 0.25847<br><.0001 | 0.38687<br><.0001 | 0.16754<br><.0001 | 0.08575<br><.0001 | 0.14585<br><.0001 | 1.00000 | 0.14201<br><.0001 | 0.09372<br><.0001 |
| **S2_Pro** | 0.27686<br><.0001 | 0.12594<br><.0001 | 0.25617<br><.0001 | 0.06233<br>0.0003 | -0.00506<br>0.7692 | 0.14201<br><.0001 | 1.00000 | 0.31279<br><.0001 |
| **S3_Pro** | 0.26356<br><.0001 | 0.20090<br><.0001 | 0.27212<br><.0001 | -0.00405<br>0.8141 | -0.00516<br>0.7647 | 0.09372<br><.0001 | 0.31279<br><.0001 | 1.00000 |

Note: Since 28 pairwise comparisons are made, the significance threshold should be considered 0.05/28=0.0018 after Bonferroni correction.

Nonsignificant correlations –indicating independent distribution of positions of proline polymorphisms in histone H1 residues between the compared populations- according to this threshold are highlighted on the upper half of the table. The proportion of non-significant correlations:

For intraspecific comparisons: 0/16.

For interspecific comparisons: 6/12.

Notice that correlations are always positive when they are significant.
